# Excess mortality attributable to the 2025 Iberian Peninsula blackout

**DOI:** 10.1038/s41467-026-75581-w

**Published:** 2026-07-20

**Authors:** Garyfallos Konstantinoudis, Julien Riou

**Affiliations:** 1 Grantham Institute for Climate Change and the Environment, Imperial College London, London, UK; 2 Department of Epidemiology and Health Systems, Unisanté, Center for Primary Care and Public Health & University of Lausanne, Lausanne, Switzerland

**Keywords:** Epidemiology, Interdisciplinary studies

## Abstract

On 28 April 2025, a widespread power outage affected mainland Portugal and Spain for around ten hours, causing major disruptions and eight reported deaths. Health impacts of blackouts range from direct effects, such as medical equipment failure, to indirect effects such as disrupted healthcare. Here we show that the 2025 Iberian blackout is associated with increased mortality in Spain, but not in Portugal. We find little evidence of excess mortality on the day of the event itself. In Spain, however, mortality rises over the next two days (+167 deaths; 95% credible interval: +28 to +300; +2.4% relative increase), particularly among women aged 85+. We also observe regional differences, but their drivers remain uncertain and may include variation in outage severity, underlying vulnerabilities, or local health-system and infrastructure conditions. These findings highlight the underestimated health burden of blackouts and the need for improved preparedness in a changing climate.

## Abstract (web summary)

The authors find that the 2025 Iberian blackout was linked to increased deaths in Spain over the following two days, especially among women aged 85 and older, but found no clear excess mortality in Portugal.

## Introduction

On 28 April 2025, electric power was interrupted across mainland Portugal and Spain for approximately ten hours, causing major societal disruptions and seven confirmed fatalities in Spain and one in Portugal^1,2^. The event highlights the growing vulnerability of European power grids. Europe has previously experienced large-scale blackouts, including the Italy-Switzerland outage in 2003, the storm-induced Münsterland blackout in 2005, and the Adriatic coast outage in 2024^3^. Such events are expected to become more frequent as European grids face mounting challenges in balancing supply and demand. These challenges stem from large-scale integration of renewables, declining fossil fuel capacity, increased exposure to cyberattacks, and the rising likelihood of extreme weather events-such as heatwaves, cold spells, floods, and storms–driven by climate change. Despite their growing frequency, the public health impacts of blackouts remain poorly quantified, particularly in terms of mortality risks associated with disrupted care, infrastructure failures, and cascading societal effects.

Power outages can affect health through a range of mechanisms. Injuries and fatalities can occur when households use alternative light, heat or power sources during outages, which can result in carbon monoxide poisoning from fuel-burning heaters or generators in enclosed or poorly ventilated spaces, and fires associated with unattended candles or improvised indoor heating and cooking practices^4,5^. These pathways accounted for four of the eight officially reported deaths during the 2025 Iberian blackout^1^, Disruptions to traffic systems, such as dark intersections or road lighting failures, can increase the risk of accidents^6^. Power failures can also disrupt healthcare services, delay emergency care, and pose serious risks to people who depend on electricity-powered medical devices^7^. Broader impacts, such as failures in water, sanitation, transport, and food systems, may further increase health risks, especially for vulnerable groups^8^. When blackouts coincide with extreme weather events, like heatwaves or cold spells, the health burden can escalate through heatstroke, hypothermia, and cardiovascular stress^9^.

Previous studies have modelled the impact of different blackout events on health^6,10,11^. Most studies have defined blackout exposure as a dichotomous variable^6,10^. However, this approach may suffer from limited statistical power, because the number of blackout days is typically small, and the computation of attributable mortality implicitly interprets the estimated association as causal, which requires strong and explicit justifications (e.g., via directed acyclic graphs and careful adjustment for confounding). A recent study examining the 2025 blackout, focusing on Spain, instead used historical data and a generalised additive model to predict the mortality that would have occurred in the absence of the blackout, thereby adopting a counterfactual framework^11^. This approach has several appealing features: the number of deaths can, in principle, be validated through cross–validation, and the method does not rely on specific assumptions about the exposure-response relationship. This counterfactual approach has several advantages: the number of deaths can be validated, through cross-validation, and the method does not rely on assumptions related to the exposure-response relationship. However, that application did not report model validation, did not account for spatial autocorrelation and excluded the COVID–19 pandemic period from the training set^11^. Ignoring spatial correlation can lead to underestimated uncertainty, while removing the COVID–19 years, rather than explicitly modelling their effects, may fail to capture recent mortality trends and variability.

We present a systematic assessment of the acute mortality toll of the 2025 Iberian blackout, with a focus on age, sex, and regional inequalities. We develop and validate 3 Bayesian spatiotemporal models and quantify the expected mortality across Portugal and Spain under the counterfactual of no blackout event. The models adjusted for key determinants of mortality, including temperature, the COVID-19 pandemic and public holidays, as well as seasonal and long-term temporal trends and regional dependences in daily death rates. By comparing observed all-cause mortality during and after the blackout to counterfactual expectations based on historical trends, we aim to capture the overall impact of the event, while disentangling individual and regional vulnerabilities.

## Results

### Mortality trends and model validation

We used all-cause mortality by day of death and population data from 22 contiguous regions across Portugal and Spain, covering a combined population of approximately 55 million (Online Supplement Table S1). Online Supplement Figure S1 shows the annual mortality rates per 1,000 population across NUTS2 regions between 2010 and 2024, with the highest rates occurring during the COVID-19 pandemic. We trained three Bayesian models using historical data from 2010 to 2024 to predict expected mortality for the first months of 2025, excluding the week of the blackout (28 April-4 May 2025). We evaluated model performance and selected the specification that best reproduced daily mortality patterns by age group ( < 65, 65-84, and  > 84 years), sex (male, female), and region (NUTS2 level; see Online Supplement Figures S2-S3). Using the best-performing model, we then predicted expected mortality for the week of the blackout, allowing us to quantify deviations from baseline. All results reported in the following sections are based on the best-performing model.

### Country-level patterns across the different lags

We found limited evidence of significant excess all-cause mortality on the day of the blackout itself (lag0) in either country, with an estimated -32 excess deaths (95% CrI: -71 to +4) in Portugal and +14 excess deaths (95% CrI: -59 to +80) in Spain, Table 1 and Online Supplement Figure S4. To account for potential delayed effects, we examined excess mortality over different, a priori selected, lag periods following the blackout. In Portugal, the results remained inconclusive: estimated excess deaths were +6 (95% CrI: -65 to +73) when considering deaths occurring within 0 to 2 days (lag0-2) after the event, and -19 (95% CrI: -154 to +94) when considering a longer lag window of in total 7 days (lag0-6). In contrast, in Spain, we found strong evidence of excess mortality within 2 days after the blackout, with an estimated +167 excess deaths (95% CrI: +28 to +300) compared to the counterfactual scenario without blackout. The 95% credible interval of +28 to +300 excess deaths reflects the range of effects that remain reasonably compatible with the data and model, acknowledging substantial uncertainty in the exact magnitude of the excess mortality. This represents a relative increase of +2.4% (95% CrI: +0.4% to +4.3%) (Online Supplement Table S2). However, we found no excess mortality in the week following the blackout.

**Table 1:** Estimates of excess all-cause deaths linked to the 2025 Iberian blackout of 28 April 2025 in Portugal and Spain by age group and sex, and with different time lags (0 days, only on the day of the blackout; 0-2 days, adding the next 2 days; and 0-6 days, adding the next 6 days)

		Portugal		
Age	Sex	Lag 0 days	Lag 0-2 days	Lag 0-6 days
65 <	Males	+7 (-3, +17)	+25 (+7, +42)	+16 (-9, +41)
65 <	Females	-3 (-11, +4)	-4 (-17, +7)	-9 (-29, +9)
65 <	Total	+5 (-8, +16)	+21 (-1, +41)	+7 (-28, +39)
65-84	Males	-5 (-23, +10)	0 (-35, +31)	-7 (-66, +42)
65-84	Females	-2 (-18, +11)	+3 (-25, +30)	-11 (-60, +36)
65-84	Total	-8 (-32, +14)	+3 (-40, +43)	-17 (-92, +48)
> 84	Males	-14 (-30, +1)	+3 (-26, +32)	26 (-27, +75)
> 84	Females	-14 (-34, +6)	-21 (-57, +17)	-34 (-107, +35)
> 84	Total	-28 (-54, -4)	-17 (-62, +27)	-10 (-95, +77)
Total	Males	-12 (-38, +12)	+28 (-15, +72)	+34 (-48, +109)
Total	Females	-20 (-46, +5)	-23 (-70, +26)	-52 (-142, +35)
Total	Total	-32 (-71, +4)	+6 (-65, +73)	-19 (-154, +94)

		Spain		
65 <	Males	0 (-21, +18)	+21 (-15, +53)	-10 (-64, +42)
65 <	Females	+9 (-6, +22)	-1 (-27, +25)	-26 (-68, +12)
65 <	Total	+8 (-17, +32)	+18 (-25, +59)	-36 (-103, +25)
65-84	Males	-10 (-44, +23)	+7 (-65, +76)	-12 (-142, +124)
65-84	Females	0 (-28, +25)	43 (-14, +96)	-6 (-113, +93)
65-84	Total	-12 (-57, +33)	+50 (-38, +140)	-26 (-185, +142)
> 84	Males	-4 (-32, +24)	+2 (-56, +60)	+5 (-115, +110)
> 84	Females	+22 (-17, +54)	+98 (+21, +167)	+124 (-37, +265)
> 84	Total	+17 (-30, +61)	+99 (+6, +190)	+127 (-70, +306)
Total	Males	-15 (-64, +34)	+27 (-68, +127)	-20 (-204, +159)
Total	Females	+29 (-21, +72)	+139 (+39, +228)	+92 (-108, +271)
Total	Total	+14 (-59, +80)	+167 (+28, +300)	+72 (-202, +319)

### Country-level patterns over time

**Fig. 1:**
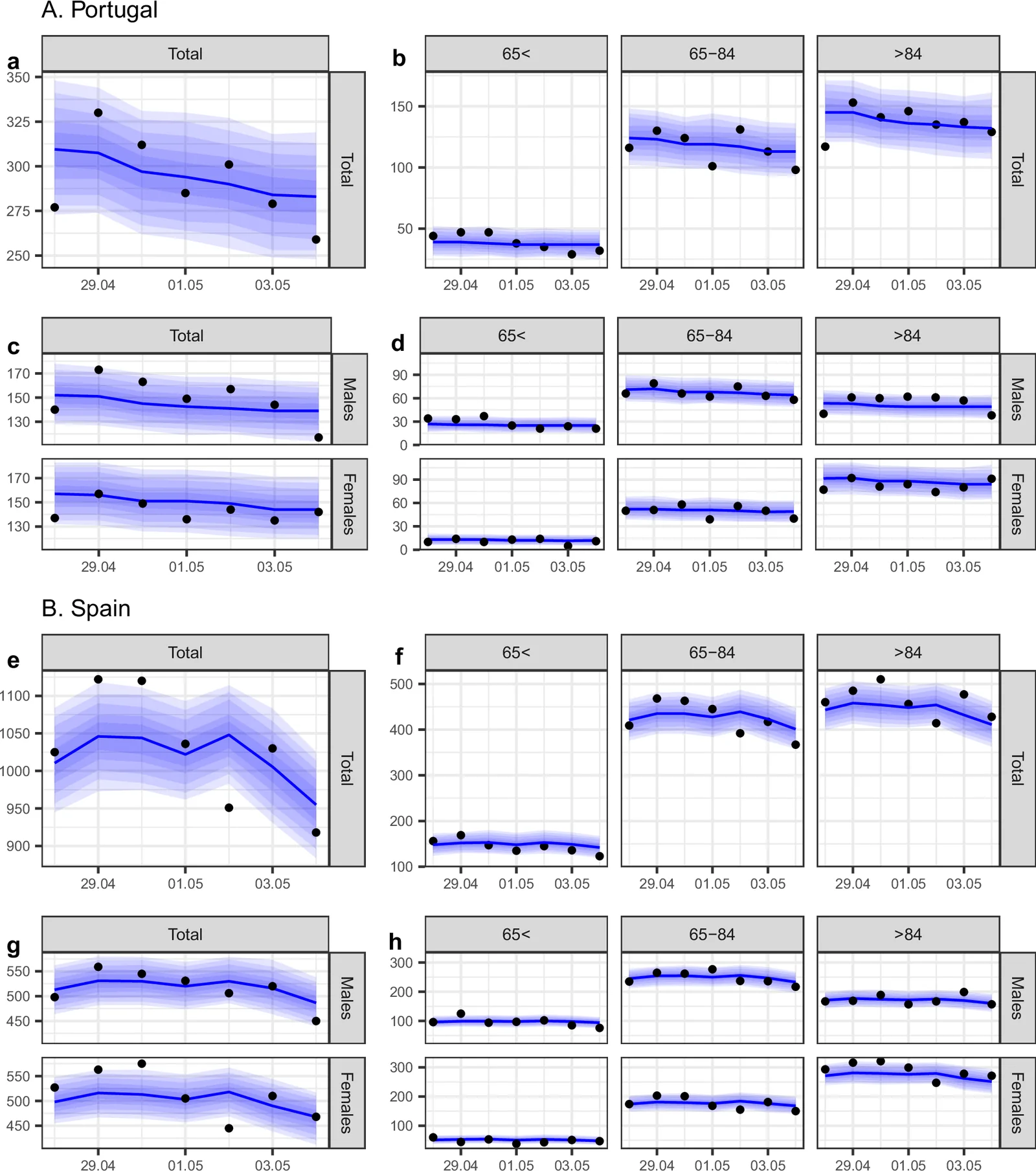
Observed and expected daily all-cause deaths during the week of the 2025 Iberian blackout in Portugal and Spain. The blue line shows the median expected number of daily all-cause deaths, the shaded blue ribbons show posterior credible intervals, and the black dots show the observed deaths. Estimates are shown for 28 April 2025 and the following six days, overall and stratified by age group and sex. Panels a–d refer to Portugal: panel **a** shows total deaths overall, panel **b** shows deaths by age group, panel **c** shows deaths by sex, and panel **d** shows deaths by age and sex group. Panels e–h refer to Spain: panel **e** shows total deaths overall, panel **f** shows deaths by age group, panel **g** shows deaths by sex, and panel **h** shows deaths by age and sex group. The different shades of blue represent posterior intervals corresponding to the 2.5%, 5%, 10%, 20%, 30%, 70%, 80%, 90%, 95%, and 97.5% percentiles of the posterior distribution.

Figure 1 shows the range of counterfactual deaths (blue ribbon) alongside the observed mortality counts (black dots) across countries, overall (panels a and e) and stratified by age (panels b and f), sex (panels c and g), and age–sex groups (panels d and h). We observed weak evidence of reduced mortality among individuals aged 85 years and older on the day of the blackout in Portugal, with an estimated -28 deaths (95% CrI: -54 to -4), Table 1, followed by increased mortality in the next two days, Fig. 1a. In aggregate, we found some evidence of increased mortality among people younger than 65 for lags 0 to 2 days (+21; 95% CrI: -1 to +41), primarily driven by males (+25; 95% CrI: +7 to +42). In Spain, there was limited evidence of excess mortality during the day of the blackout across all age and sex groups, but observed number of deaths exceeded the 95% credible interval of the expected counts on the following two days, Fig. 1b. The evidence towards increased mortality was strong for lags of 0 to 2 days for people older than 85 years, with an estimate of +99 (95% CrI: +6 to +190), primarily driven by females (+98 ; 95% CrI: +21 to +167), Table 1. Over the entire week of the blackout (lags 0 to 6 days), we found no evidence of an increased mortality.

### Sub-national level patterns

**Fig. 2:**
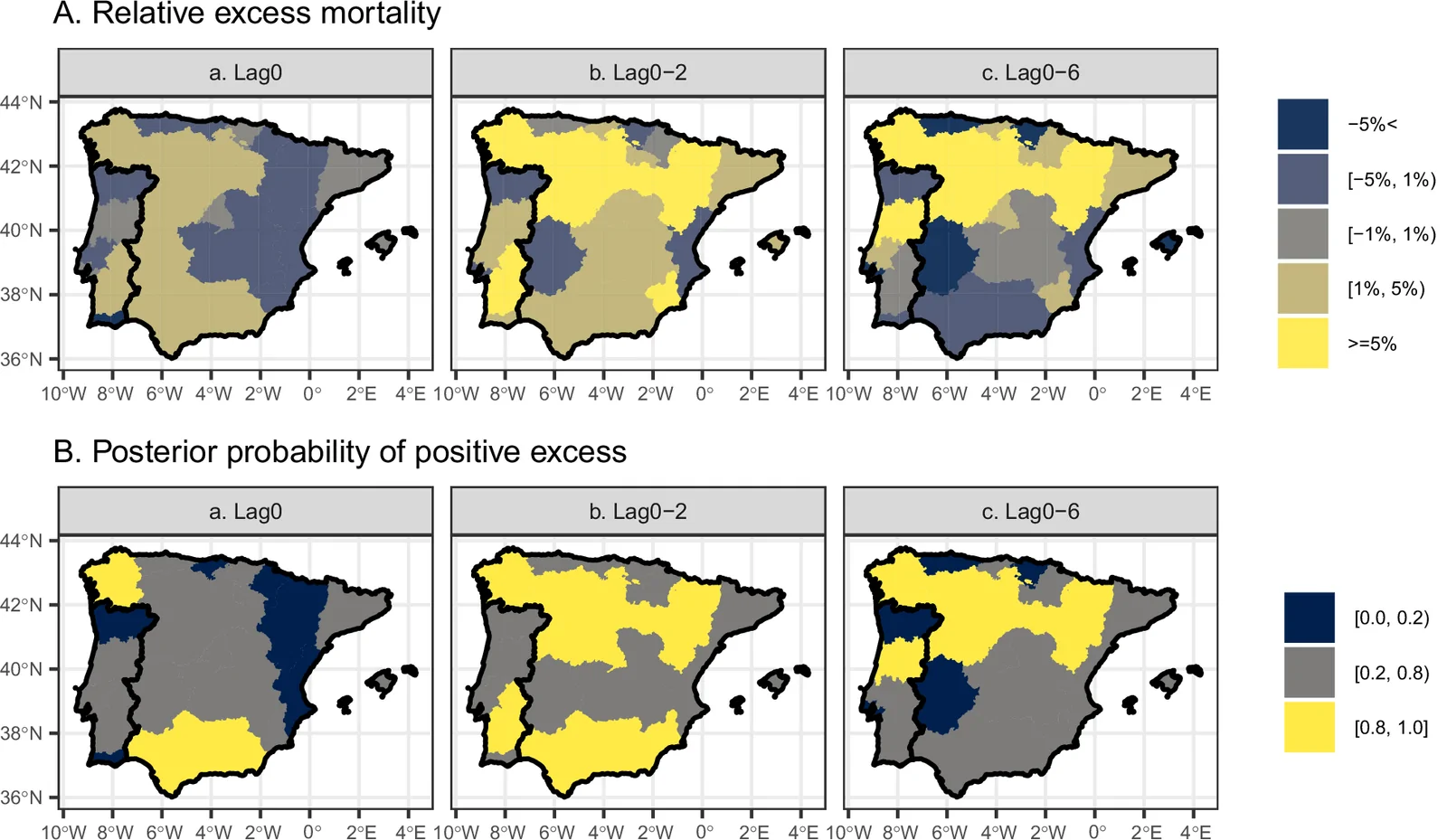
Regional estimates of excess all-cause mortality following the 2025 Iberian blackout in Portugal and Spain. **A** shows the median relative excess all-cause mortality by NUTS-2 region, and **B** shows the posterior probability that excess all-cause deaths were greater than zero. In both panels, subpanel **a** shows estimates for lag 0 (the day of the blackout only), **b** shows estimates for lag 0--2 (the blackout day and the following 2 days), and **c** shows estimates for lag 0--6 (the blackout day and the following 6 days).

The geographical distribution of excess mortality across the regions of both countries revealed additional insights (Fig. 2). The top panel of Fig. 2 shows the percentage relative change in excess mortality, while the bottom panel displays the posterior probability that this excess is positive, reflecting the strength of evidence. In Portugal, the reduced mortality observed on the day of the blackout was mostly driven by the Algarve and Norte regions (north and south of Portugal in Fig. 2A). However, accounting for lagged effects over the subsequent 2 or 6 days, evidence of increased mortality emerged in the central regions of Alentejo and Centro. In Spain, excess mortality on the day of the blackout was limited to the northwestern region of Galicia and the southern region of Andalusia (Fig. 2). When considering lags of 0 to 2 days, the pattern became more geographically widespread (Fig. 2). There are some indications that excess mortality even persisted up to 6 days after the blackout in some areas of the northern half of Spain (Fig. 2).

### Sensitivity analysis

Our initial choice for modelling space across the 3 model candidates did not account for spatial autocorrelation; a decision driven by the very few regions available in Portugal (6 regions). In the first sensitivity analysis, we relaxed this assumption for the best performing model and modelled space using a reparametrisation of the Besag-York-Mollie model^12^. The results were identical, see Online Supplement Figure S5. To assess robustness of the results with respect to the different choices made to model the non-linear and lagged effect of temperature^13^, we tested another common parametrisation used in the literature^14^ and found that the results were identical, Online Supplement Figure S6. We last investigated the association between regional excess mortality and socioeconomic context, as proxied by gross domestic product (GDP) per capita. NUTS2-level GDP per capita for Spain and Portugal was obtained from Eurostat in 2023 and linked to the corresponding regional estimates of excess mortality. Visual inspection of scatterplots and correlation patterns across the different lags indicated no clear evidence of a systematic relationship between GDP per capita and the magnitude of excess mortality, Online Supplement Figure S7-8.

## Discussion

In this multi-country study we examined excess mortality associated with the 2025 Iberian blackout. Our results indicate that blackout-induced disruptions were associated with moderate increases in mortality, primarily observed or reported one to two days after the event. Based on these data alone, it remains unclear whether these excess deaths occurred on the day of the blackout and were identified with reporting delays, or whether they reflect lagged health consequences stemming from the disruptions. Nonetheless, our findings suggest that the total mortality burden, though moderate, substantially exceeds the seven officially confirmed deaths reported in Spain^1^. Given that such directly attributable fatalities are typically identified and reported promptly, it is plausible that the remaining 160 excess deaths reflect indirect impacts of the blackout, such as delays in emergency care, disruptions in chronic disease management, or environmental exposures.

A few studies have documented short-term spikes in all-cause mortality during major power outages, on the day or in the subsequent few days^4,15^. The 2003 Northeast U.S.A. blackout was linked to an increase of about 122% of accidental deaths and of 25% of non-accidental deaths, with older adults (65–74 years old) experiencing the largest mortality surge ^6^. Other smaller power outages in New York City between 2002 and 2014 have also been associated with mortality increases of 2 to 6% in the following 1–3 days^10^. A 2019 typhoon-related blackout in Tokyo saw all-cause deaths increase by about 11% in the first three days post-event^15^. Such mortality impacts can be exacerbated when blackouts coincide with extreme weather events, which may explain the somewhat lower estimates reported for the 2025 Iberia blackout, which occurred in relatively mild weather conditions. Our results are consistent with a 2025 blackout study in Spain^11^. The estimated excess deaths among women at lag 0–2 are compatible across studies, with that paper reporting 125 excess deaths (95% CI  − 3,  + 254) and our analysis yielding 139 excess deaths (95% CrI  + 39,  + 228). In contrast to their findings, which suggested the largest effects in the 65–84 years age group, we identified people aged  > 85 years as the most affected. However, the respective uncertainty intervals overlap, indicating that these apparent differences by age group should be interpreted cautiously.

Our reported estimates of excess mortality varied across countries, regions or demographic groups, suggesting that the impact of the blackout was variable across contexts and settings. In Spain, the concentration of excess mortality in females aged 85 or older is consistent with disruptions affecting outpatient medical services, domiciliary nursing and care homes, settings in which power outages have been linked with increased short-term mortality^16^. The absence of comparable effects in older males could be partly explained by the underlying age structure, as women are disproportionately represented in the oldest age groups (Online Supplement Figure S9), where frailty and dependence on electrically powered services are more common^17^. We did not find comparable effects in Portugal, which may reflect differences in blackout duration, baseline frailty, health-system and infrastructure conditions, and organisational features of elderly care, including preparedness measures such as backup power capacity. Indeed, Portugal has a highly centralised and integrated public long-term care system^18^, whereas the Spanish system is characterized by decentralisation and marked territorial disparities^19^. We also found weak evidence of excess mortality among working-age males in Portugal blue that could reflect random variation or a modest rise in accidental deaths associated with the blackout. Additional research with cause-of-death data and high-resolution exposure metrics is needed to elucidate these mechanisms.

Some of the regional differences could be explained by variation in blackout duration. The blackout began at 12:33 CEST on 28 April 2025 and led to a near–total loss of supply in mainland Spain and Portugal. System–level reports indicate that electricity supply in mainland Spain was largely restored during the evening of 28 April, with approximately 99% of demand recovered by 07:00 CEST and full restoration by 11:00 CEST on 29 April. According to the Spanish transmission system operator, parts of northern Spain and Andalusia were reconnected earlier, around 17:00 CEST on 28 April, whereas other areas of the peninsula remained without power for longer. In mainland Portugal, around half of the population had power back by late evening on 28 April and full restoration was achieved in the early hours of 29 April. However, detailed restoration times are not available at sub–national level, which prevents a direct assessment of whether within–country differences in blackout duration account for the observed regional heterogeneity in health impacts.

The excess mortality peak observed at lag 0-2 days in Spain, particularly among women aged  > 84 years, is no longer apparent when we consider the entire week after the blackout. A plausible explanation is a harvesting (or mortality displacement) mechanism, whereby very frail individuals die slightly earlier than they would have in the absence of the blackout. Under this mechanism, an early spike in deaths can be followed by a period (or a day, Fig. 1) of lower–than–expected mortality, so that cumulative excess mortality over a longer window (lag 0-6 days) is attenuated or even returns towards the null. In addition, estimates over longer lags inherently include more days that were only weakly, if at all, affected by the blackout, which introduces statistical noise and dilutes the signal from the short, intense exposure period.

The excess mortality framework proposed here is based on advanced Bayesian statistical modelling, which robustly captures direct and indirect mortality effects attributable to the blackout. The proposed methodology is readily replicable in other geographical and temporal settings for assessing the impacts of blackout events. A similar Bayesian framework has been developed beyond blackout scenarios. The authors developed hierarchical bayesian spatiotemporal models to quantify the COVID-19 mortality toll during the first year of the pandemic^20,21^. Ensemble hierarchical bayesian spatiotemporal models have been used to quantify the mortality toll of climate extremes such as heatwaves in Switzerland during summer 2022^22^ and tropical cyclones in the US^23^. Overall, this methodology can be a valuable tool for monitoring a wide range of extremes in the climate change era, including droughts, wildfires, and infectious disease outbreaks. We provide a thorough validation of the proposed model tailored to the specific context of daily all-cause mortality in Spain and Portugal in the spring of 2025, which underscores the high reliability of our model predictions in terms of bias and uncertainty. Finally, our results are based on updated population estimates, and account for the specific effects of the COVID-19 pandemic on all-cause mortality during the epidemic waves of 2020-2024.

However, we face several limitations, including potential residual confounding due to unmeasured variables such as past or concurrent public health emergencies. This may lead to bias or increased uncertainty in the computation of expected deaths, which may in turn lead to limiting the sensitivity of our assessment of excess deaths. Likewise, the temporal and geographical granularity of our analytical framework may limit the detection of small-scale or localized effects. This analysis is limited to one event, which strongly limits the generalisability of our findings as blackouts differ in duration, population affected and contexts. Further research comparing multiple events is needed to disentangle the mechanisms and interactions that determine the public health impact of blackouts, including infrastructure failures, healthcare system resilience, demographic vulnerability, and concurrent environmental or societal stressors. We did not incorporate air pollution in our predictive model. Including air pollution as a covariate in the prediction model would be expected to block part of the blackout effect operating through changes in air pollution. If blackout events modify emissions and therefore air–pollution levels, air-pollution would lie on the causal pathway between blackout and mortality; conditioning on such a mediator would potentially bias the estimated total effect of the blackout. Although respiratory virus circulation can influence mortality, the blackout occurred outside the usual respiratory syncytial virus season in Spain and Portugal^24^, and surveillance data indicated no unusual respiratory syncytial virus, influenza or COVID-19 activity during April 2025, making concurrent epidemic activity an unlikely explanation for the observed patterns and suggesting limited gains in predictive performance from including these covariates. In addition, given the one-week-ahead prediction horizon, mortality dynamics captured by the second-order random walk would likely account for much of the effect of any ongoing respiratory virus activity. Last, our study lacks detailed data on the causes of death, which would have allowed insights into the sources of the observed variation in excess deaths.

In conclusion, our analysis underscores the important health consequences of blackouts and power outages, particularly in the elderly, emphasising the need for strengthened resilience and planning in healthcare infrastructure. While the decarbonisation of power systems is essential for climate mitigation, the April 2025 blackout reveals the fragility introduced by high renewable penetration without parallel investments in grid resilience. The health consequences of this event highlight the urgent need for policies that integrate renewable energy growth with robust system balancing, storage, and emergency health preparedness.

## Methods

This study uses routinely collected, population-level observational data on mortality, population, temperature and national holidays. No statistical method was used to predetermine sample size, as the analysis includes all available observations for the study period and regions considered. No data were excluded from the analyses. Randomisation was not applicable because the study did not involve any intervention or allocation of participants or regions to exposure groups. Blinding was also not applicable in the conventional experimental sense, because the analysis was conducted on pre-existing aggregated data without treatment allocation or prospective outcome assessment.

### Outcome data

We retrieved daily all-cause mortality data at the NUTS2 level for the period 2010-2025 from the National Statistics Institute in Portugal, and from the National Centre of Epidemiology at the Carlos III Health Institute, the Daily Monitoring Mortality System, the National Statistics Institute, and the Ministry of Justice in Spain. The number of deaths from all causes was available by sex and age. We grouped age into three categories:  < 65, 65–84, and  > 84 years.

### Population data

Annual population figures for Portugal were obtained from the National Statistics Institute for the years 2011-2023, with estimates referring to December 31 of each year. For Spain, annual population data were obtained from the National Statistics Institute for the years 2010–2024, with figures consistently available for January 1 of each year. To estimate population counts for missing years, we applied a two-stage linear interpolation approach as previously described^20^. Briefly, we first used linear regression to estimate annual population figures for missing years (i.e., 2009, 2010, 2024, and 2025 for Portugal; and 2025 for Spain). We then performed linear interpolation to derive daily population estimates between 2010 and May 2025. Interpolation was conducted by age group, sex, and NUTS2 region. The resulting estimates were used as denominators in our analyses.

### Covariates

To support the prediction of daily counterfactual expected mortality, we included temperature and national holidays as covariates^25^. Temperature data were obtained from the ERA5 reanalysis dataset provided by the Copernicus Climate Data Store^26^. For each centroid of the 0.25^∘^ × 0.25^∘^ grid cells, we calculated the daily mean temperature for the period 2010-2025. These values were then aggregated to the NUTS2 level using population weights derived from the WorldPop dataset (https://www.worldpop.org), as they are available on a finer grid to match with the temperature data. We used the year 2018 for population weighting as the midpoint of our analysis. As mortality from all causes can vary during national holidays, we also included a binary variable indicating whether a given day is a public holiday, accounting for the regional holidays in Spain.

COVID-19 waves had a marked impact on mortality during parts of the 2020-2024 period. However, excluding the entire 2020-2024 period from the training set would introduce other challenges, particularly in capturing long-term mortality trends across age and sex groups. We therefore adopted a pragmatic approach, excluding only the weeks with substantial COVID-19 impact while retaining other weeks from this period to help anchor and characterize temporal mortality trends. Specifically, we used data on laboratory-confirmed COVID-19 deaths in Portugal and Spain from Our World In Data^27^, and excluded from the training set any weeks with more than 100 such deaths in either country.

### Statistics & Reproducibility

We considered three models to estimate the daily expected number of all-cause deaths, separately for each age and sex group and for the different countries (for simplicity we omit these indices in the following). Let *Y*_*slmt*_ denote the number of all-cause deaths and *P*_*slmt*_ the population in the *s*-th NUTS2 region on the *l*-th day of the week, *m*-th week of the year, and *t*-th year:

1 $${Y}_{slmt} \sim {{{\rm{Poisson}}}}\left({\mu }_{slmt}{P}_{slmt}\right)$$

2 $$\log ({\mu }_{slmt})={\beta }_{0}+{\beta }_{1}{X}_{1smt}+f({r}_{slmt},k)+{z}_{s}+{w}_{l}+{q}_{m}+{\delta }_{*}$$

where *β*_0_ is the intercept, *β*_1_ represents the effect of public holidays (accounting for regional holidays as well), *f*(*r*_*slmt*_, *k*) captures the non-linear effect of temperature at lag k on all-cause mortality, *z*_*s*_ models spatial autocorrelation between NUTS2 regions, *w*_*l*_ captures day-of-week effects, *q*_*m*_ captures seasonality by week of the year, and δ_*_ represents the long-term temporal trend.

We modelled δ_*_ using three specifications with increasing complexity, allowing us to examine a range of different temporal trends (random noise, linear, and non-linear). Model 1 assigns an unstructured random effect on calendar time to model temporal trends, model 2 adds a linear trend in calendar time on model 1 and model 3 models calendar time using a structured temporal random effect: Model 1: δ_*_ = *u*_*mt*_, where *u*_*mt*_ is assigned an i.i.d. normal prior with no structure on the covariance matrix.

Model 2: δ_*_ = *u*_*mt*_ + *β*_2_*X*_2*mt*_, where *X*_2*mt*_ denotes the week and the year and *β*_2_ is a fixed linear trend.

Model 3: δ_*_ = *v*_*mt*_, where *v*_*mt*_ follows a second-order random walk process.

The non-linear effect *f*( ⋅ ) of mean daily temperature *r*_*slmt*_ at lag *k* is modelled using distributed lag non-linear models (DLNMs)^13^. For the choice of temperature we specified a quadratic B-spline with 5 degrees of freedom for temperature, with knots placed by default at equally spaced value in the space of the predictor, in line with previous studies^28^. We used the same parametrisation for the lag dimension and constructed the crossbasis matrix by combining these functions. The choice of 21 is justified as the effects of cold temperatures are expected to be prolonged^13^. Spatial autocorrelation *z*_*s*_ is modelled with an i.i.d. normal prior without spatial structure, given the relatively large size of NUTS2 regions.

The models were considered in a Bayesian framework and fitted using Integrated Nested Laplace Approximation^29^. We assigned an improper uniform prior to the intercept. For fixed effects, we used normal priors with mean zero and precision 0.001. These also include the coefficients of the crossbasis function, which account for the non-linear and prolonged effects of temperature on health. For the hyperparameters governing the random effects’ precision, we applied penalised complexity (PC) priors^30^. PC priors shrink toward a parsimonious base model (typically corresponding to no random effect), with the degree of shrinkage controlled through interpretable probability statements on the variance parameter^30^. Using such probability statements, we calibrated the priors to ensure it is unlikely that relative risks exceed exp(2) due solely to spatial or temporal variation.

We trained the model using data from 2010 to 2025 (up to and including 27 April 2025) and generated predictions of area-level daily mortality for the week of the blackout, spanning from 28 April to 4 May 2025. To summarise the results, we retrieved samples from the posterior predictive distribution of daily expected mortality by age group, sex, and NUTS2 region for Spain and Portugal. We report the daily observed number of deaths during the blackout week alongside the corresponding posterior distribution of the expected number of deaths had the blackout not occurred. We also report the median and 95% Credible Intervals (CrI) of the cumulative number of excess deaths (observed minus expected) for three, a priori selected, time windows: (i) the day of the blackout (lag0), (ii) the blackout day plus two subsequent days (lag0-2), and (iii) the blackout day plus six subsequent days, to capture the entire week (lag0-6). Finally, we retrieved posterior estimates of the relative (percent) increase in mortality, i.e., the increase relative to the expected number of deaths had the blackout not occurred, by NUTS2 region.

### Cross-validation

To select the best-performing of the three considered model formulations, we performed a leave-the-last-week-out cross-validation. Briefly, we first defined the training set as the years 2010 to 2024, and the testing set as the first week of 2025. We then extended the training set to include the first week of 2025 and updated the testing set to the second week of 2025. This iterative procedure was repeated for both countries until all weeks up to and including 27 April 2025, one day before the blackout, were included. To compare model performance across the different cross-validation sets, we calculated three metrics: (i) bias, defined as the difference between the predicted and observed values; (ii) square root of mean squared error (MSE), calculated as square root of the average of squared differences between predicted and observed values; and (iii) coverage probability, defined as the proportion of observed values falling within the 95% credible interval of the predictions.

### Sensitivity analyses

In the first sensitivity analysis, we replaced the unstructured prior (iid) used to model spatial autocorrelation with a reparameterisation of the Besag-York-Mollié model^12^. To assess the robustness of our results to alternative temperature parameterisations, we specified a quadratic B-spline with knots placed at the 10th, 75th, and 90th percentiles of the region-specific temperature distribution. For the lag dimension, we employed a cubic natural spline with three knots positioned at equally spaced values on the logarithmic scale, assuming a lag period of 21 days^14^. Finally, we examined the association between regional excess mortality and socioeconomic context, proxied by gross domestic product (GDP) per capita. NUTS2-level GDP per capita data for Spain and Portugal were obtained from Eurostat (2023) and linked to the corresponding regional estimates of excess mortality. We then plotted the median posterior excess mortality alongside scaled GDP per capita for Spain and Portugal to assess whether regional differences could be explained by variations in GDP.

### Reporting summary

Further information on research design is available in the Nature Portfolio Reporting Summary linked to this article.

## Supplementary information

Supplementary Information

Reporting Summary

Transparent Peer Review file

## Data Availability

Population data for Spain are publicly available from the Instituto Nacional de Estadística (https://www.ine.es/jaxiT3/Tabla.htm?t=56945&L=0). Mortality data for Spain are not publicly available because they are protected under data protection regulations, but access may be requested from the National Centre of Epidemiology at the Carlos III Health Institute, subject to the relevant approvals. Mortality and population data for Portugal can be obtained from the Instituto Nacional de Estatística (https://www.ine.pt/). Temperature data are publicly available from the ERA5-Land dataset (https://cds.climate.copernicus.eu/datasets/reanalysis-era5-land?tab=download). Data on national holidays were obtained from the Nager.Date database (https://date.nager.at/).

## Code availability

All models were fitted using the Integrated Nested Laplace Approximation (INLA: version INLA_24.12.09) using its R software (R version 4.4.2) interface^29^. Code for reproducing the analysis can be retrieved from GitHub (https://github.com/jriou/blackout-burden) and Zenodo (https://zenodo.org/records/20643229).
