# Supplementary material for: Excess mortality attributable to the 2025 Iberian Peninsula blackout
Source: Nat Commun. 2026 Jul 20;17:8805. doi: 10.1038/s41467-026-75581-w (PMC13493769; doi:10.1038/s41467-026-75581-w)
Supplement: Supplementary file 1 [file 41467_2026_75581_MOESM1_ESM.pdf]

Online Supplement: Excess deaths attributable to the 2025 Iberian Peninsula blackout

Garyfallos Konstantinoudis[1,*] and Julien Riou[2]

[1]Grantham Institute for Climate Change and the Environment, Imperial College London, London, UK

[2]Department of Epidemiology and Health Systems, Unisanté, Center for Primary Care and Public Health & University of Lausanne

[*]Corresponding author, email: g.konstantinoudis@imperial.ac.uk

List of Supplementary Tables

S1 Total and mean number of deaths, mean population and mortality rate during 2010–2024 and area [in km^2] across the different NUTS2 regions in Portugal and Spain. 5

S2 Estimates of percentage relative excess all-cause deaths linked to the 2025 Iberian blackout of 28 April 2025 in Portugal and Spain by age group and sex, and with different time lags (0 days, only on the day of the blackout; 0-2 days, adding the next 2 days; and 0-6 days, adding the next 6 days). The results are based on Model 3. 6

List of Supplementary Figures

S1 Annual mortality rates per 1,000 population by NUTS2 regions in Portugal and Spain during 2010-2024. 7

S2 Cross validation results: Bias, coverage probability and square root of mean squared error across the different age and sex group for models 1, 2 and 3 in **Portugal**. All models include an intercept, the effect of public holidays, a non-linear and lagged effect of temperature, a spatial autocorrelation term, a day of the week effect and a seasonality term. Model 1 (M1) uses an unstructured random effect on calendar time to model temporal trends, Model 2 (M2) adds a linear trend in calendar time on model 1 and Model 3 (M3) models calendar time using a structured random effect. The error bars show the median and 95% credible intervals, whereas the boxplots show the median and interquartile range of the posterior distribution of the estimates. 8

S3 Cross validation results: Bias, coverage probability and square root of mean squared error across the different age and sex group for models 1, 2 and 3 in **Spain**. All models include an intercept, the effect of public holidays, a non-linear and lagged effect of temperature, a spatial autocorrelation term, a day of the week effect and a seasonality term. Model 1 (M1) uses an unstructured random effect on calendar time to model temporal trends, Model 2 (M2) adds a linear trend in calendar time on model 1 and Model 3 (M3) models calendar time using a structured random effect. The error bars show the median and 95% credible intervals, whereas the boxplots show the median and interquartile range of the posterior distribution of the estimates. 9

S4 Estimates of excess all-cause deaths (median and 95% Credible Intervals) linked to the 2025 Iberian blackout of 28 April 2025 in Portugal and Spain by age group and sex, and with different time lags (0 days, only on the day of the blackout, 2 days, and 1 week after). 10

S5 Median expected daily all-cause deaths (solid blue line) with 95% credible interval (blue ribbon) and corresponding observations of all-cause deaths (black dots) on the week of the 2025 Iberian blackout (28 April 2025 and the following six days), overall and stratified by age group and sex. The different shades of blue represent different percentiles of the posterior distribution (2.5%, 5%, 10%, 20%, 30%, 70%, 80%, 90%, 95% and 97.5%). There results refer to sensitivity analysis 1, replacing the unstructured random effect in space with a BYM2 prior [1]. 11

S6 Median expected daily all-cause deaths (solid blue line) with 95% credible interval (blue ribbon) and corresponding observations of all-cause deaths (black dots) on the week of the 2025 Iberian blackout (28 April 2025 and the following six days), overall and stratified by age group and sex. The different shades of blue represent different percentiles of the posterior distribution (2.5%, 5%, 10%, 20%, 30%, 70%, 80%, 90%, 95% and 97.5%). There results refer to sensitivity analysis 2, assessing robustness to another parametrisation of the temperature crossbasis function [2]. 12

S7 Scaled GDP (Gross Domestic Product) per NUTS2 region as of 2023 in Portugal and Spain. . . . 13

S8 Scatterplot of scaled GDP (Gross Domestic Product) per NUTS2 region as of 2023 versus median relative excess all-cause mortality by lag period and country. 14

S9 Number of deaths by age and sex across Portugal and Spain during 2010–2025. 15

Table S1: Total and mean number of deaths, mean population and mortality rate during 2010–2024 and area [in km^2] across the different NUTS2 regions in Portugal and Spain.

NUTS2	Number of deaths Total	Mean	Population	Mortality rate per 1,000	Area in km^2
Portugal					
Alentejo	111,975	7,465	484,580	15.41	27,239
Algarve	77,698	5,180	459,556	11.27	4,974
Área Metropolitana de Lisboa	425,492	28,366	2,855,491	9.93	2,844
Centro	313,639	20,909	1,667,830	12.54	23,233
Norte	525,329	35,022	3,625,496	9.66	21,256
Oeste e Vale do Tejo	160,695	10,713	841,141	12.74	9,197
Spain					
Andalucía	1,075,186	71,679	8,437,572	8.50	87,577
Aragón	187,832	12,522	1,331,353	9.41	47,660
Cantabria	70,062	4,670	586,126	7.97	5,325
Castilla y León	337,767	22,518	2,443,829	9.21	94,076
Castilla-La Mancha	285,226	19,015	2,067,634	9.20	79389
Cataluña	985,929	65,729	7,617,136	8.63	32,073
Comunidad Foral de Navarra	84,823	5,655	650,731	8.69	10,385
Comunidad Valenciana	674,300	44,953	5,038,073	8.92	23, 277
Comunidad de Madrid	626,156	41,744	6,580,400	6.34	8,010
Extremadura	156,185	10,412	1,077,873	9.66	41,604
Galicia	475,719	31,715	2,722,591	11.65	29,558
Illes Balears	126,326	8,422	1,146,807	7.34	5,010
La Rioja	36,650	2,443	318,256	7.68	5,045
País Vasco	316,036	21,069	2,193,659	9.60	7,202
Principado de Asturias	197,776	13,185	1,035,540	12.73	10,597
Región de Murcia	167,938	11,196	1,493,617	7.50	11,310

Table S2: Estimates of percentage relative excess all-cause deaths linked to the 2025 Iberian blackout of 28 April 2025 in Portugal and Spain by age group and sex, and with different time lags (0 days, only on the day of the blackout; 0-2 days, adding the next 2 days; and 0-6 days, adding the next 6 days). The results are based on Model 3.

Age	Sex	Lag0	Pr	Lag0-2	Pr	Lag0-6	Pr
Portugal							
65<	Males	4.24% (-1.55%, 10.23%)	0.91	14.20% (3.66%, 25.95%)	1.00	8.94% (-4.41%, 26.62%)	0.87
65<	Females	-3.14% (-11.32%, 4.71%)	0.20	-4.76% (-17.17%, 10.00%)	0.23	-10.47% (-27.36%, 13.24%)	0.16
65<	Total	1.89% (-2.98%, 6.62%)	0.74	7.96% (-0.34%, 17.39%)	0.96	2.64% (-9.33%, 16.74%)	0.65
65-84	Males	-1.11% (-4.70%, 2.23%)	0.24	0.00% (-6.70%, 6.86%)	0.47	-1.47% (-12.34%, 9.84%)	0.40
65-84	Females	-0.64% (-4.96%, 3.33%)	0.34	0.84% (-6.46%, 9.32%)	0.58	-3.10% (-14.86%, 11.69%)	0.32
65-84	Total	-1.01% (-3.70%, 1.72%)	0.22	0.36% (-4.59%, 5.39%)	0.54	-2.05% (-10.17%, 6.27%)	0.32
>84	Males	-3.83% (-7.92%, 0.27%)	0.03	0.87% (-6.48%, 10.15%)	0.58	7.37% (-6.65%, 24.68%)	0.83
>84	Females	-2.38% (-5.39%, 0.99%)	0.06	-3.48% (-8.72%, 3.06%)	0.14	-5.55% (-15.60%, 6.43%)	0.19
>84	Total	-2.86% (-5.40%, -0.40%)	0.01	-1.78% (-6.16%, 2.91%)	0.23	-1.03% (-9.03%, 8.74%)	0.42
Total	Males	-1.17% (-3.59%, 1.24%)	0.16	2.78% (-1.48%, 7.73%)	0.88	3.37% (-4.40%, 11.67%)	0.80
Total	Females	-1.90% (-4.21%, 0.50%)	0.07	-2.19% (-6.33%, 2.66%)	0.18	-4.94% (-12.43%, 3.63%)	0.11
Total	Total	-1.57% (-3.33%, 0.19%)	0.04	0.29% (-2.97%, 3.69%)	0.57	-0.92% (-7.01%, 4.82%)	0.38
Spain							
65<	Males	0.00% (-3.01%, 2.70%)	0.46	3.00% (-2.03%, 8.04%)	0.87	-1.46% (-8.66%, 6.64%)	0.36
65<	Females	2.49% (-1.68%, 6.34%)	0.86	-0.28% (-6.98%, 7.33%)	0.45	-7.18% (-16.84%, 3.70%)	0.10
65<	Total	0.78% (-1.63%, 3.05%)	0.71	1.75% (-2.30%, 5.91%)	0.80	-3.39% (-9.25%, 2.54%)	0.15
65-84	Males	-0.59% (-2.46%, 1.39%)	0.27	0.41% (-3.52%, 4.67%)	0.58	-0.69% (-7.59%, 7.73%)	0.43
65-84	Females	0.00% (-2.19%, 2.11%)	0.47	3.52% (-1.08%, 8.22%)	0.92	-0.48% (-8.40%, 8.17%)	0.45
65-84	Total	-0.39% (-1.88%, 1.12%)	0.30	1.68% (-1.23%, 5.02%)	0.86	-0.87% (-5.88%, 5.04%)	0.40
>84	Males	-0.32% (-2.58%, 2.10%)	0.39	0.17% (-4.32%, 5.33%)	0.52	0.42% (-8.71%, 10.05%)	0.53
>84	Females	1.14% (-0.88%, 2.96%)	0.86	5.15% (1.03%, 9.33%)	0.99	6.52% (-1.80%, 15.06%)	0.93
>84	Total	0.56% (-0.94%, 2.00%)	0.75	3.20% (0.18%, 6.43%)	0.98	4.09% (-2.12%, 10.47%)	0.89
Total	Males	-0.42% (-1.72%, 0.94%)	0.28	0.73% (-1.80%, 3.68%)	0.70	-0.54% (-5.35%, 4.61%)	0.42
Total	Females	0.83% (-0.58%, 2.10%)	0.87	3.98% (1.06%, 6.78%)	1.00	2.63% (-2.92%, 8.16%)	0.81
Total	Total	0.21% (-0.81%, 1.13%)	0.65	2.35% (0.38%, 4.31%)	0.99	1.02% (-2.73%, 4.63%)	0.70

Figure S1: Annual mortality rates per 1,000 population by NUTS2 regions in Portugal and Spain during 2010-2024.

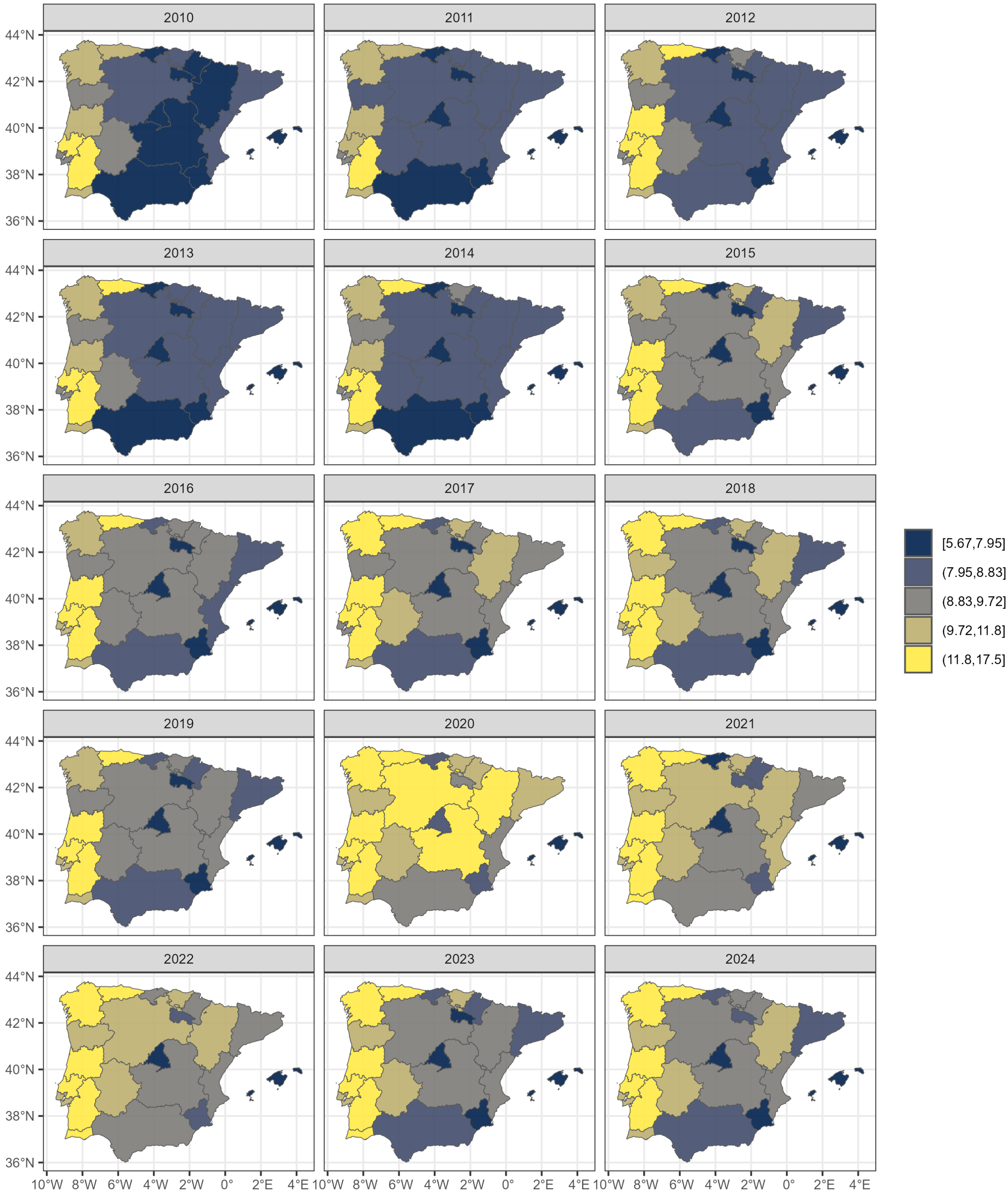

Figure S2: Cross validation results: Bias, coverage probability and square root of mean squared error across the different age and sex group for models 1, 2 and 3 in **Portugal**. All models include an intercept, the effect of public holidays, a non-linear and lagged effect of temperature, a spatial autocorrelation term, a day of the week effect and a seasonality term. Model 1 (M1) uses an unstructured random effect on calendar time to model temporal trends, Model 2 (M2) adds a linear trend in calendar time on model 1 and Model 3 (M3) models calendar time using a structured random effect. The error bars show the median and 95% credible intervals, whereas the boxplots show the median and interquartile range of the posterior distribution of the estimates.

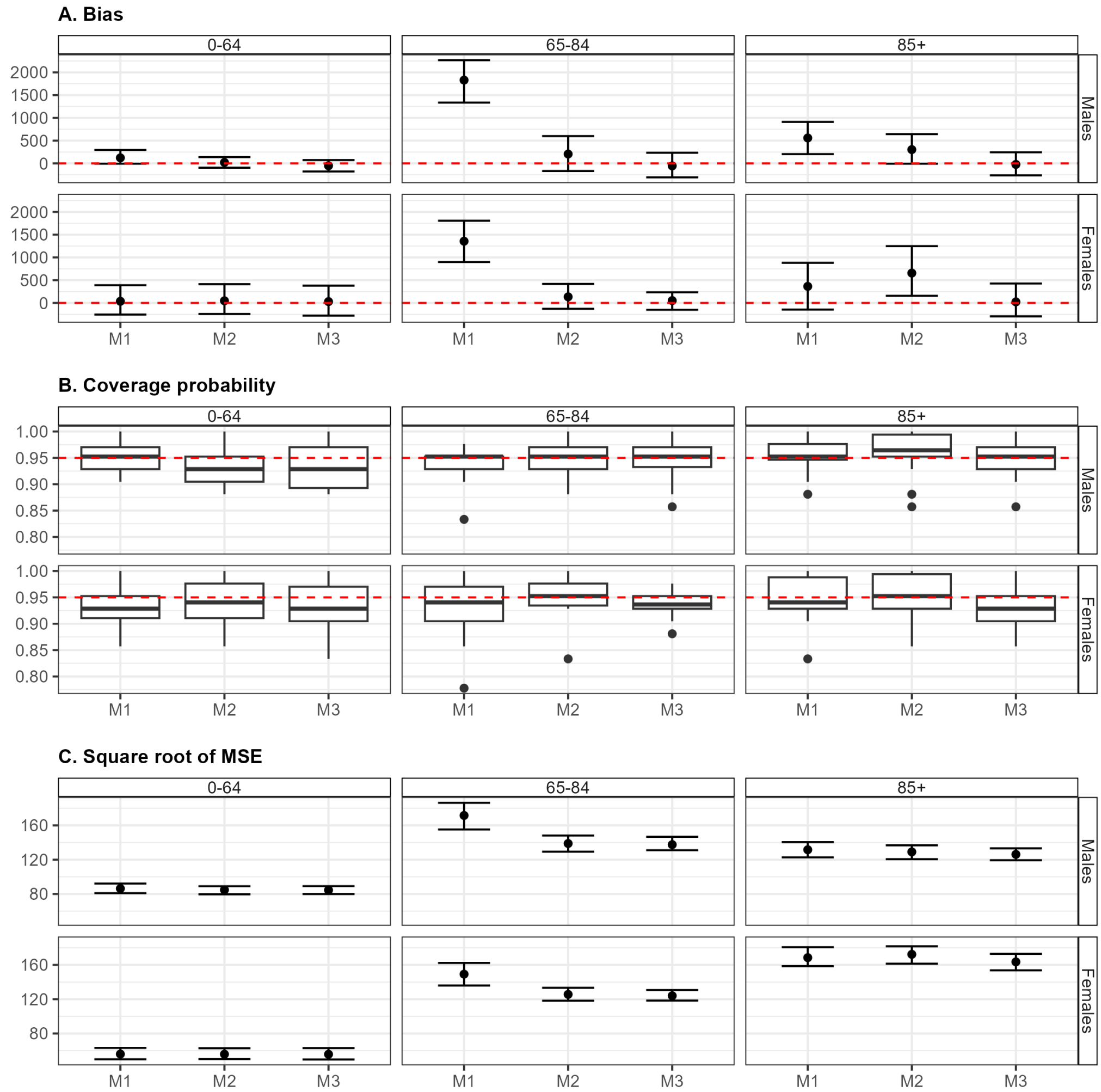

Figure S3: Cross validation results: Bias, coverage probability and square root of mean squared error across the different age and sex group for models 1, 2 and 3 in **Spain**. All models include an intercept, the effect of public holidays, a non-linear and lagged effect of temperature, a spatial autocorrelation term, a day of the week effect and a seasonality term. Model 1 (M1) uses an unstructured random effect on calendar time to model temporal trends, Model 2 (M2) adds a linear trend in calendar time on model 1 and Model 3 (M3) models calendar time using a structured random effect. The error bars show the median and 95% credible intervals, whereas the boxplots show the median and interquartile range of the posterior distribution of the estimates.

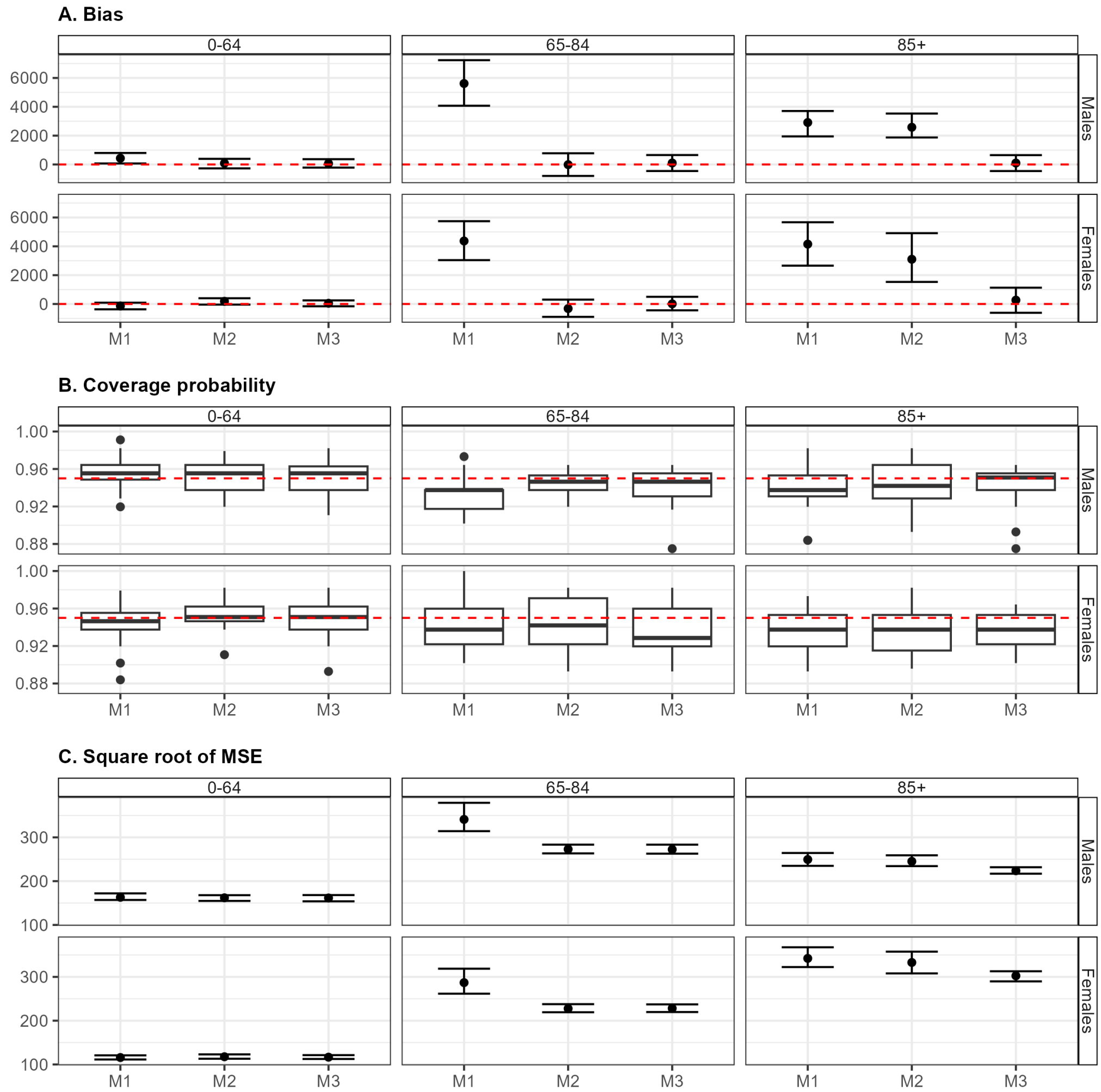

Figure S4: Estimates of excess all-cause deaths (median and 95% Credible Intervals) linked to the 2025 Iberian blackout of 28 April 2025 in Portugal and Spain by age group and sex, and with different time lags (0 days, only on the day of the blackout, 2 days, and 1 week after).

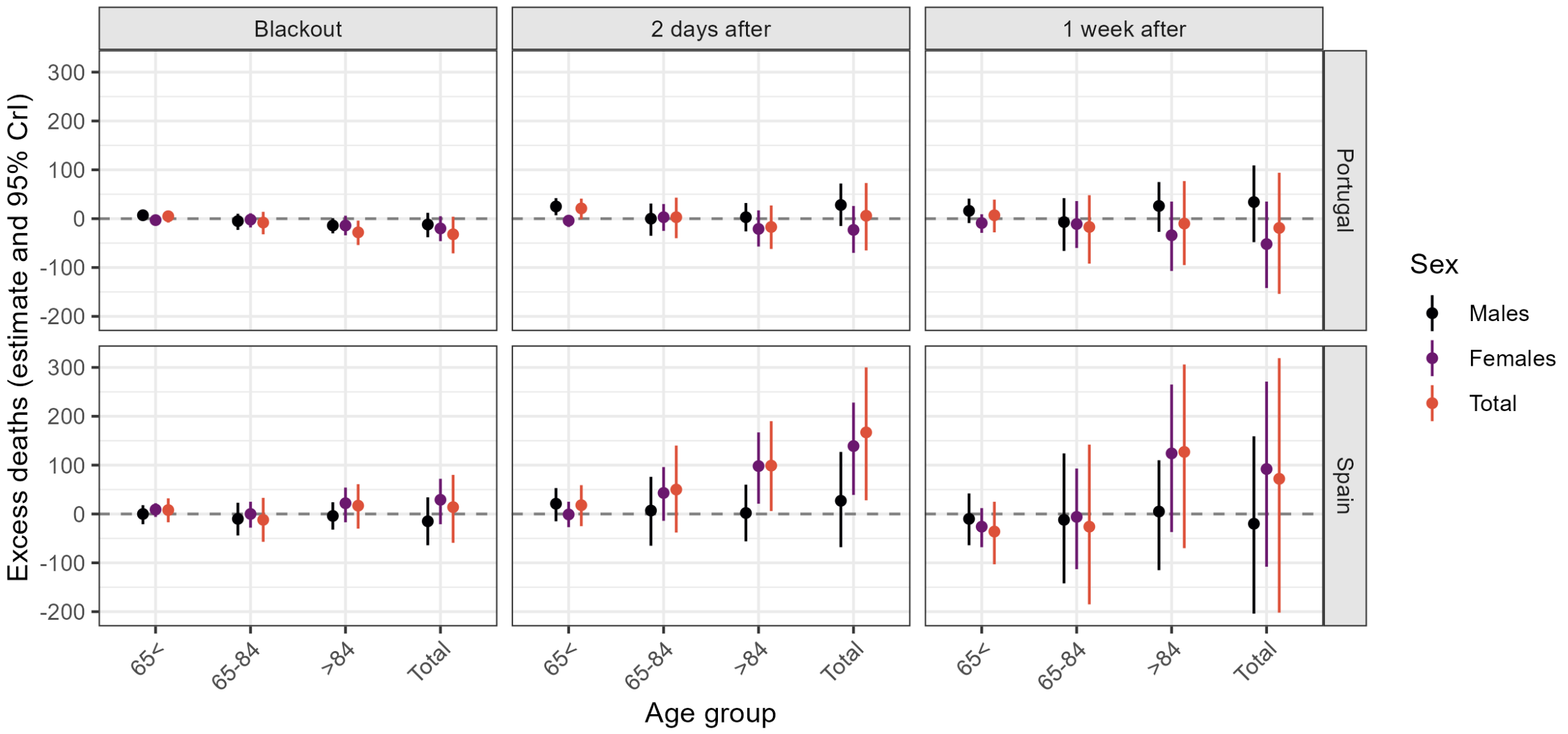

Figure S5: Median expected daily all-cause deaths (solid blue line) with 95% credible interval (blue ribbon) and corresponding observations of all-cause deaths (black dots) on the week of the 2025 Iberian blackout (28 April 2025 and the following six days), overall and stratified by age group and sex. The different shades of blue represent different percentiles of the posterior distribution (2.5%, 5%, 10%, 20%, 30%, 70%, 80%, 90%, 95% and 97.5%). There results refer to sensitivity analysis 1, replacing the unstructured random effect in space with a BYM2 prior [1].

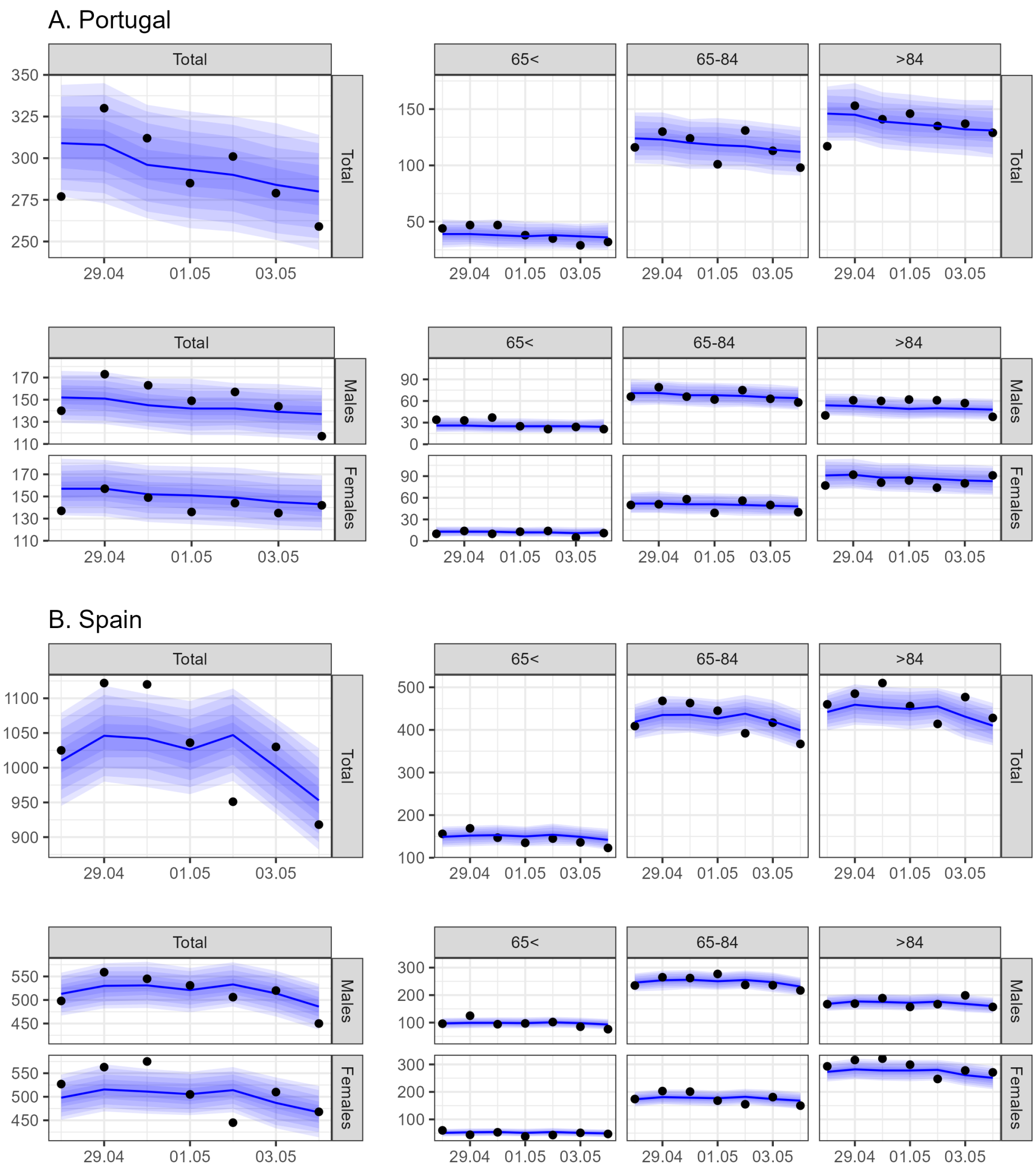

Figure S6: Median expected daily all-cause deaths (solid blue line) with 95% credible interval (blue ribbon) and corresponding observations of all-cause deaths (black dots) on the week of the 2025 Iberian blackout (28 April 2025 and the following six days), overall and stratified by age group and sex. The different shades of blue represent different percentiles of the posterior distribution (2.5%, 5%, 10%, 20%, 30%, 70%, 80%, 90%, 95% and 97.5%). There results refer to sensitivity analysis 2, assessing robustness to another parametrisation of the temperature crossbasis function [2].

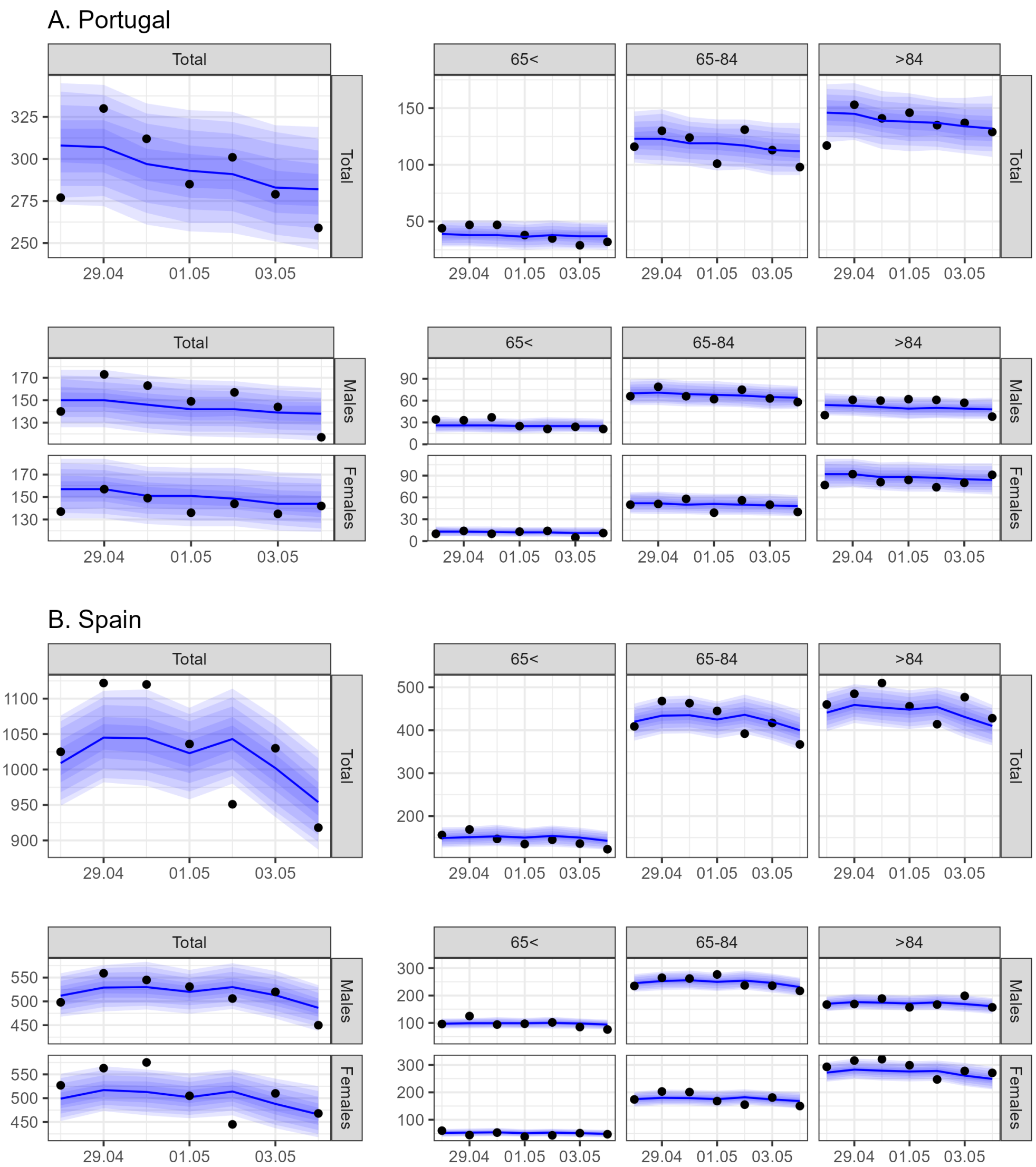

Figure S7: Scaled GDP (Gross Domestic Product) per NUTS2 region as of 2023 in Portugal and Spain.

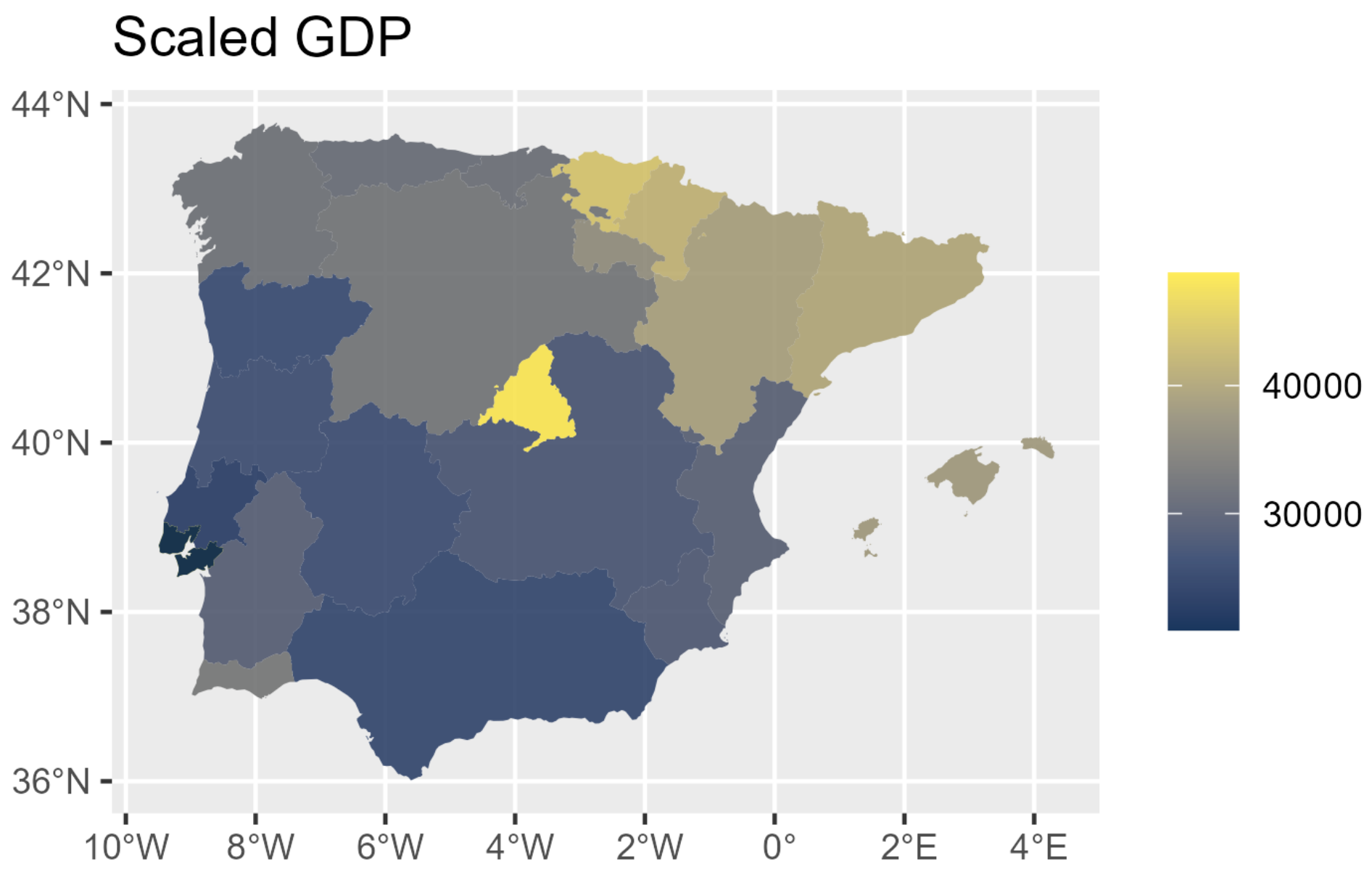

Figure S8: Scatterplot of scaled GDP (Gross Domestic Product) per NUTS2 region as of 2023 versus median relative excess all-cause mortality by lag period and country.

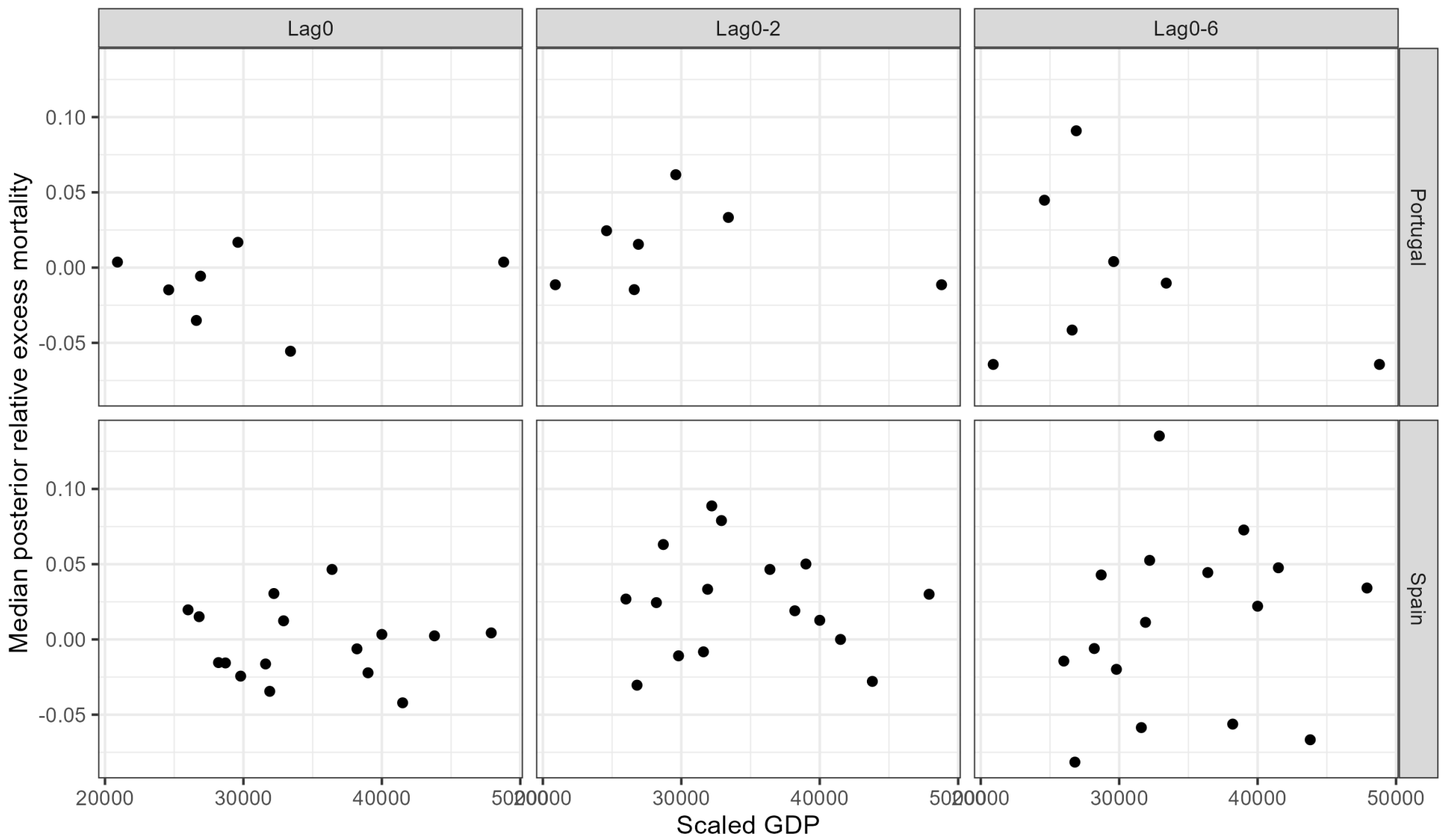

Figure S9: Number of deaths by age and sex across Portugal and Spain during 2010–2025.

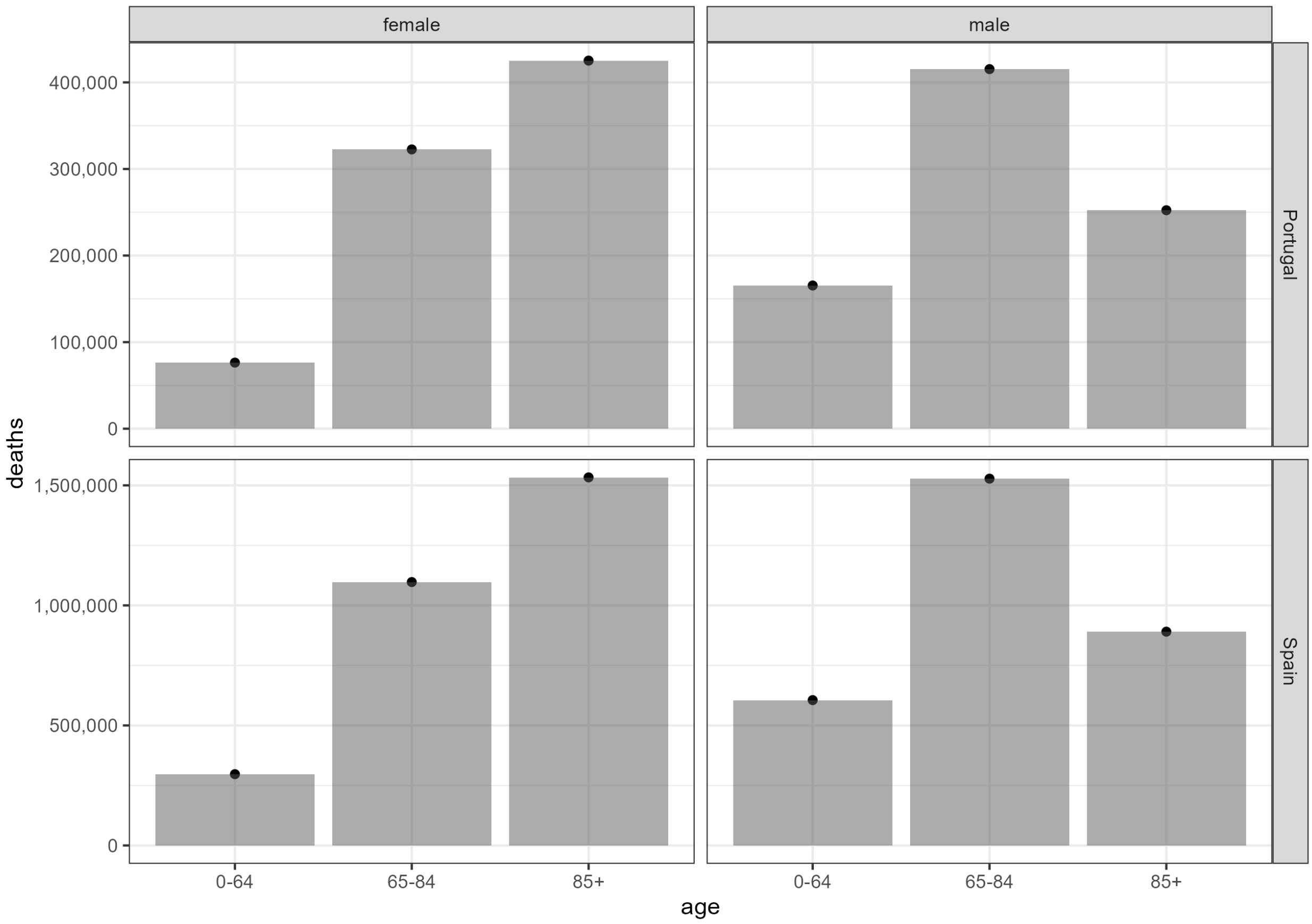

References

[1] Andrea Riebler, Sigrunn H Sørbye, Daniel Simpson, and Håvard Rue. An intuitive Bayesian spatial model for disease mapping that accounts for scaling. *Statistical Methods in Medical Research*, 25(4):1145–1165, 2016.

[2] Pierre Masselot, Malcolm Mistry, Jacopo Vanoli, Rochelle Schneider, Tamara Iungman, David Garcia-Leon, Juan-Carlos Ciscar, Luc Feyen, Hans Orru, Aleš Urban, et al. Excess mortality attributed to heat and cold: a health impact assessment study in 854 cities in europe. *The Lancet Planetary Health*, 7(4):e271–e281, 2023.
