# Supplementary material for: Excess mortality attributable to the 2025 Iberian Peninsula blackout
Source: Nat Commun. 2026 Jul 20;17:8805. doi: 10.1038/s41467-026-75581-w (PMC13493769; doi:10.1038/s41467-026-75581-w)
Supplement: Supplementary file 2 [file 41467_2026_75581_MOESM2_ESM.pdf]

nature portfolio

Corresponding author(s): Garyfallos Konstantinoudis

Last updated by author(s): Jun 10, 2026

Reporting Summary

Nature Portfolio wishes to improve the reproducibility of the work that we publish. This form provides structure for consistency and transparency in reporting. For further information on Nature Portfolio policies, see our Editorial Policies and the Editorial Policy Checklist.

Statistics

For all statistical analyses, confirm that the following items are present in the figure legend, table legend, main text, or Methods section.

n/a	Confirmed	
☐	☒	The exact sample size (*n*) for each experimental group/condition, given as a discrete number and unit of measurement
☒	☐	A statement on whether measurements were taken from distinct samples or whether the same sample was measured repeatedly
☒	☐	The statistical test(s) used AND whether they are one- or two-sided<br>*Only common tests should be described solely by name; describe more complex techniques in the Methods section.*
☐	☒	A description of all covariates tested
☒	☐	A description of any assumptions or corrections, such as tests of normality and adjustment for multiple comparisons
☐	☒	A full description of the statistical parameters including central tendency (e.g. means) or other basic estimates (e.g. regression coefficient) AND variation (e.g. standard deviation) or associated estimates of uncertainty (e.g. confidence intervals)
☒	☐	For null hypothesis testing, the test statistic (e.g. F, t, r) with confidence intervals, effect sizes, degrees of freedom and P value noted<br>*Give P values as exact values whenever suitable.*
☐	☒	For Bayesian analysis, information on the choice of priors and Markov chain Monte Carlo settings
☒	☐	For hierarchical and complex designs, identification of the appropriate level for tests and full reporting of outcomes
☒	☐	Estimates of effect sizes (e.g. Cohen's *d*, Pearson's *r*), indicating how they were calculated

Our web collection on statistics for biologists contains articles on many of the points above.

Software and code

Policy information about availability of computer code

Data collection	All data processing was performed using the statistical software R.
Data analysis	All data analysis was conducted using the statistical software R (R version 4.4.2) and the INLA package (INLA_24.12.09), a package for Bayesian analysis freely available at https://www.r-inla.org/. The code used is also online available at (https://github.com/jriou/blackout-burden).

For manuscripts utilizing custom algorithms or software that are central to the research but not yet described in published literature, software must be made available to editors and reviewers. We strongly encourage code deposition in a community repository (e.g. GitHub). See the Nature Portfolio guidelines for submitting code & software for further information.

Data

Policy information about availability of data

All manuscripts must include a data availability statement. This statement should provide the following information, where applicable:

- Accession codes, unique identifiers, or web links for publicly available datasets
- A description of any restrictions on data availability
- For clinical datasets or third party data, please ensure that the statement adheres to our policy

Population data in Spain can be retrieved from the Instituto Nacional de Estadistica in Spain (https://www. ine.es/). Mortality data in Spain can be retrieved after request from the National Centre of Epidemiology at the Carlos III Health Institute. Mortality and population data in Portugal can be retrieved from the Instituto Nacional de Estadistica in Portugal (https://www.ine.pt/).

Research involving human participants, their data, or biological material

Policy information about studies with human participants or human data. See also policy information about sex, gender (identity/presentation), and sexual orientation and race, ethnicity and racism.

Reporting on sex and gender	To investigate potential inequalities in impact, the main analysis includes sex-stratified analyses to assess whether the blackout disproportionately affected one sex relative to the other.
Reporting on race, ethnicity, or other socially relevant groupings	Information on race, ethnicity, or other socially relevant groupings was not available in the mortality datasets used for this study in Spain and Portugal. As a result, we were unable to examine whether the impact of the blackout differed across these population groups.
Population characteristics	Between 2010 and 2024, we retrieved a total of 5,951,033 deaths in Spain, of which 51% were among males (N=3,023,776). By age group, there were 902,413 deaths among people aged 0–64 years, 2,624,958 among those aged 65–84 years, and 2,423,662 among those aged 85 years and older. Over the same period, we retrieved 1,657,134 deaths in Portugal, of which 50% were among males (N=833,078). By age group, there were 241,717 deaths among people aged 0–64 years, 737,921 among those aged 65–84 years, and 677,496 among those aged 85 years and older.
Recruitment	N/A. The study uses nationwide data on all-cause mortality in Spain and Portugal. In both countries, death registration is mandatory.
Ethics oversight	The study is about secondary, aggregate anonymised data so no additional ethical permission is required.

Note that full information on the approval of the study protocol must also be provided in the manuscript.

Field-specific reporting

Please select the one below that is the best fit for your research. If you are not sure, read the appropriate sections before making your selection.

☐ Life sciences ☒ Behavioural & social sciences ☐ Ecological, evolutionary & environmental sciences

For a reference copy of the document with all sections, see nature.com/documents/nr-reporting-summary-flat.pdf

Behavioural & social sciences study design

All studies must disclose on these points even when the disclosure is negative.

Study description	The study uses quantitative methods. We applied Bayesian spatiotemporal models to historical all-cause mortality data in Portugal and Spain during 2010-2025 by sex and age group (65<, 65-84, 84>) to estimate daily and subnational all-cause mortality for the blackout week (28 April–4 May 2025), had the blackout not occurred. We then compared it with the observed mortality and retrieved the mortality impact due to the blackout.
Research sample	We performed the analysis in Spain and Portugal, as these were the two countries affected by the blackout event.
Sampling strategy	N/A. We used all reported deaths available for analysis.
Data collection	Mortality data in Spain can be retrieved after request from the National Centre of Epidemiology at the Carlos III Health Institute. Mortality data in Portugal is online available (https://www.ine.pt/).
Timing	Mortality data from 2010 to 2025 were included in this study. The dataset was accessed and downloaded in May 2025. The source database is continuously updated with data for more recent time periods.
Data exclusions	We used all relevant data for our research question.
Non-participation	N/A. Our data is population based counts of mortality and population based on the national registries and interpolation methods of data from censuses, migration, deaths and birth registries.
Randomization	Our study is observational and not experimental. We did not use individual participants, rather the complete data on mortality and population in Portugal and Spain.

Reporting for specific materials, systems and methods

We require information from authors about some types of materials, experimental systems and methods used in many studies. Here, indicate whether each material, system or method listed is relevant to your study. If you are not sure if a list item applies to your research, read the appropriate section before selecting a response.

Materials & experimental systems

n/a	Involved in the study
☒	☐ Antibodies
☒	☐ Eukaryotic cell lines
☒	☐ Palaeontology and archaeology
☒	☐ Animals and other organisms
☒	☐ Clinical data
☒	☐ Dual use research of concern
☒	☐ Plants

Methods

n/a	Involved in the study
☒	☐ ChIP-seq
☒	☐ Flow cytometry
☒	☐ MRI-based neuroimaging

Plants

Seed stocks	N/A
Novel plant genotypes	N/A
Authentication	N/A
