# Supplementary material for: Excess mortality attributable to the 2025 Iberian Peninsula blackout
Source: Nat Commun. 2026 Jul 20;17:8805. doi: 10.1038/s41467-026-75581-w (PMC13493769; doi:10.1038/s41467-026-75581-w)
Supplement: Supplementary file 3 [file 41467_2026_75581_MOESM3_ESM.pdf]

nature portfolio

Peer Review File

Excess mortality attributable to the 2025 Iberian Peninsula blackout

Corresponding Author: Dr Garyfallos Konstantinoudis

This file contains all reviewer reports in order by version, followed by all author rebuttals in order by version.

Attachments originally included by the reviewers as part of their assessment can be found at the end of this file.

Version 0:

Reviewer comments:

Reviewer #1

(Remarks to the Author)
See Reviewer Attachment

(Remarks on code availability)

Reviewer #2

(Remarks to the Author)
I co-reviewed this manuscript with one of the reviewers who provided the listed reports. This is part of the Nature Communications initiative to facilitate training in peer review and to provide appropriate recognition for Early Career Researchers who co-review manuscripts.

(Remarks on code availability)

Reviewer #3

(Remarks to the Author)
This manuscript focuses on the recent Iberian Blackout of 2025, which impacted broad populations and lasted up to ten hours. The authors leverage publicly available all-cause mortality data and use Bayesian time series methods to estimate the impact of the blackout on the same-day as the event, lags 0-2, and over the following week. Differences in impact by country, region, age, and sex emerge. The manuscript is well written and appropriately characterizes the findings. I have a few suggestions to improve the work.

Major
1. Lines 93-101: while I agree with some of the speculation from the authors as to why sex and age and county-specific differences were observed, this section seems too speculative for a scientific manuscript. Please present alternative reasons and cite sources for the claims made in the paragraph.
2. Modeling approach: Should any inclusion of circulating respiratory viruses be considered that might impact mortality?
3. Signals are detected and modeling performance is good, but I would emphasize the range of the credible interval further (from 28 to 300).
4. Given the observed spatial heterogeneity is there any further blackout data available to link duration of the event across space? I assume that the blackout did not last the same duration across the study region and this might help explain observed differences.

Minor

1. Abstract mentions direct effect of power outage is fire. This link is not immediately clear and should be clarified or removed from the abstract.
2. Lines 30-31: please explain how CO poisoning occurs, this is tied to improper generator use. Please describe how fires occur, is this tied to candle use?
3. Line 63: Did excess deaths over the week following the fire become inconclusive or evidence was found of no excess mortality in the week following the blackout?
4. Figure 1: why are the credible intervals multiple shades of blue?
5. Figure 2 presenting geographic heterogeneity on relative scale and probability of excess is useful
6. Supplemental Figure 1: please describe what models 1, 2, and 3 are in the figure caption.
7. Line 84: a brief description of differences in the regions of Spain would be helpful. What is different about Northern Spain, for example?
8. Line 153-159: have others used a similar approach to handle the COVID-19 pandemic effects? Please provide citations if so.

(Remarks on code availability)

Reviewer #4

(Remarks to the Author)
First of all, I would like to thank the authors for the importance of this study and its innovative methodological implementation. It is a very well-structured and explained piece of work.

The only thing I found lacking in the analysis was the income factor. In Spain, there are rural areas, even provinces, where the population has very, very low incomes, and this can make death due to this type of event more likely.

One suggestion, if possible, would be to include the income or GDP of the provinces, which would be interesting (if possible).

Thank you very much for such an interesting topic.

(Remarks on code availability)

Reviewer #5

(Remarks to the Author)
Please, see attached pdf.

(Remarks on code availability)
Repository is not accesible yet. For sure, If they make it available, it would be a pleasure to review it.

Version 1:

Reviewer comments:

Reviewer #1

(Remarks to the Author)
Based on my co-review, we feel the authors have largely addressed the comments and suggestions in the first round of review. We have only one additional request of the authors. We do not need to review the next revision, if the editors consider the revision responsive.

We had asked for region-level details such as mortality rates that may explain the heterogeneity in the mortality seen across the different regions, as provided in Figure 2 in the main paper. However, the table added to the paper provides little information, or the information is hard to match because the regions in the table do not clearly map to the regions in the figure. Two ways to make the connection cleaner would be to either include a numerical table of the results at the region level or to provide a heatmap of relevant region-level statistics in a similar format. Moreover, the table provides one data point per region for the period 2010–25. Either a year-by-year breakdown or a single data point covering only the most recent few years would be a better gauge. The authors should also present the mortality rate rather than the raw number of deaths. Additional contextual statistics in the table — for example, GDP per capita — would have been useful, but the authors have added a scatterplot of region-level excess mortality against GDP per capita (supplementary figure S7), and find no correlation pattern via visual inspection. So, this may address that.

(Remarks on code availability)

Reviewer #2

(Remarks to the Author)
I co-reviewed this manuscript with one of the reviewers who provided the listed reports. This is part of the Nature Communications initiative to facilitate training in peer review and to provide appropriate recognition for Early Career Researchers who co-review manuscripts.

(Remarks on code availability)

Reviewer #3

(Remarks to the Author)
Thank you for a strong revision. A couple small comments:

- Do nursing care facilities in Spain generally have onsite backup generators? This is common in some other high-income countries but would be helpful context. Or perhaps these are more common in Portugal.
- Regarding circulation of respiratory viruses, the new sentence is helpful. I wondered if including these data might improve model fit, as we have seen this to be the case in our large-scale epidemiologic studies.
-Abstract: "Regional differences could suggest varying healthcare resilience." This is quite speculative for an abstract. I might also include "or variation in outage severity." Or cut this and add something else.

(Remarks on code availability)
I have no reviewed the code.

Reviewer #4

(Remarks to the Author)
Accept as it is

(Remarks on code availability)

Version 2:

Reviewer comments:

Reviewer #1

(Remarks to the Author)
I co-reviewed this manuscript with one of the reviewers who provided the listed reports. This is part of the Nature Communications initiative to facilitate training in peer review and to provide appropriate recognition for Early Career Researchers who co-review manuscripts.

(Remarks on code availability)

Reviewer #3

(Remarks to the Author)
No additional comments, thank you for the additional information.

(Remarks on code availability)

Open Access This Peer Review File is licensed under a Creative Commons Attribution 4.0 International License, which permits use, sharing, adaptation, distribution and reproduction in any medium or format, as long as you give appropriate credit to the original author(s) and the source, provide a link to the Creative Commons license, and indicate if changes were made.
In cases where reviewers are anonymous, credit should be given to 'Anonymous Referee' and the source.
The images or other third party material in this Peer Review File are included in the article's Creative Commons license, unless indicated otherwise in a credit line to the material. If material is not included in the article's Creative Commons license and your intended use is not permitted by statutory regulation or exceeds the permitted use, you will need to obtain permission directly from the copyright holder.
To view a copy of this license, visit https://creativecommons.org/licenses/by/4.0/

Point by point reply

Reviewer 1

Review Report

The paper studies the effect of a 10-hour blackout across mainland Spain and Portugal. It utilizes three different Poisson regression specifications trained on the data from 2010-2025 (up until the week of the blackout) to predict mortality rates, and then chooses specification (M3) as the best of their three specifications based on the metrics of bias, coverage, and mean squared error. Authors use the predictions by this model as counterfactuals and compare them with the observed mortality. The difference in mortality is associated with the effects of the blackout. Authors disaggregate mortality rates for each country by sex, age groups (0-64, 65-85, and 85+), and by lag periods: lag 0 defined as the day of the blackout, lag 0-2 days, and lag 0-6 days.

Significance

This paper addresses a public health question with limited prior evidence: the impact of blackouts on mortality. Given that extreme climate events are increasing and have the potential to cause blackouts, it is important to understand how such events affect public health to inform efficient policy responses. Unfortunately, the study does not investigate the underlying mechanisms ("how"), focusing solely on changes in mortality rates. The paper offers a narrow empirical result in one region of the world from one particular event. Is that of sufficient impact for a broad interdisciplinary journal?

Authors' reply: Thank you for your comment. It is correct that our paper deliberately focuses on the 2025 Iberian blackout. Blackouts differ widely in duration, population affected and

contexts, so that pooling events to produce global estimates would mix heterogeneous mechanisms (e.g., flood-, heatwave- or storm-induced outages) and situations (e.g., low-, middle- and high-income countries) and yield little actionable insights. One idea could be to focus on a particular region, but very few blackouts of comparable scale occurred in western Europe in the last 20 years (i.e. Italy-Switzerland 2003, Münsterland 2005, Adriatic coast 2024). While we agree it could be valuable to produce a wider comparison of the health effects of power outages, accounting for different factors, we still believe that the 2025 Iberian blackout offers by itself interesting insights about the health risk posed by blackouts in a European context, especially as climate change increases stress on the electrical grid. We have added to following sentences to the discussion to further limit claims of generalisability, lines 192-196 on page 7:

This analysis is limited to one event, which strongly limits the generalisability of our findings as blackouts differ in duration, population affected and contexts. Further research comparing multiple events is needed to disentangle the mechanisms and interactions that determine the public health impact of blackouts, including infrastructure failures, healthcare system resilience, demographic vulnerability, and concurrent environmental or societal stressors.

Given data, the methodology is certainly replicable elsewhere. Maybe the authors can think about adding a paragraph to discuss its applicability elsewhere and for future studies.

Authors' reply: We have now added a couple of sentences discussing the generalisability of the methodology, lines 176-183 on pages 6-7:

The proposed methodology is readily replicable in other geographical and temporal settings for assessing the impacts of blackout events. A similar Bayesian framework has been developed beyond blackout scenarios. The authors developed hierarchical bayesian spatiotemporal models to quantify the COVID-19 mortality toll during the first year of the pandemic \cite{konstantinoudis2022regional, konstantinoudis2023workflow} . Ensemble hierarchical bayesian spatiotemporal models have been used to quantify the mortality toll of

climate extremes such as heatwaves in Switzerland during summer 2022 \cite{konstantinoudis2025ensemble} and tropical cyclones in the US \cite{parks2023short}. Overall, this methodology can be a valuable tool for monitoring a wide range of extremes in the climate change era, including droughts, wildfires, and infectious disease outbreaks.

In general, the narrative lacks a description of the methods used by other studies (which are referenced first in the Results, which is rather late) that have estimated mortality from power outages. The authors claim the methodology is their main novelty/contribution – if so, they should justify this against previous used methodologies. Similarly, the authors claim their method has been used in other Covid studies. Can the authors elaborate on how other studies used their method, and why that's relevant to this study

Authors' reply: We have now added a paragraph to the Introduction that elaborates on previous studies and explains how our approach compares with them (page 2, lines 41–55):

Previous studies have modelled the impact of different blackout events on health \cite{andersonLightsOutImpact2012, dominianniHealthImpactsCitywide2018a, garcia2025exploring}. Most studies have defined blackout exposure as a dichotomous variable \cite{andersonLightsOutImpact2012, dominianniHealthImpactsCitywide2018a}. However, this approach may suffer from limited statistical power, because the number of blackout days is typically small, and the computation of attributable mortality implicitly interprets the estimated association as causal, which requires strong and explicit justifications (e.g., via directed acyclic graphs and careful adjustment for confounding). A recent study examining the 2025 blackout, focusing on Spain, instead used historical data and a generalised additive model to predict the mortality that would have occurred in the absence of the blackout, thereby adopting a counterfactual framework \cite{garcia2025exploring}. This approach has several appealing features: the number of deaths can, in principle, be validated through cross-validation, and the method does not rely on specific assumptions about the exposure–response relationship . This counterfactual

approach has several advantages: the number of deaths can be validated, through cross-validation, and the method does not rely on assumptions related to the exposure–response relationship. However, that application did not report model validation, did not account for spatial autocorrelation and excluded the COVID-19 pandemic period from the training set \cite{garcia2025exploring}. Ignoring spatial correlation can lead to underestimated uncertainty, while removing the COVID-19 years, rather than explicitly modelling their effects, may fail to capture recent mortality trends and variability.

In addition, we included several sentences discussing studies that employ a similar method:

A similar Bayesian framework has been developed beyond blackout scenarios. The authors developed hierarchical bayesian spatiotemporal models to quantify the COVID-19 mortality toll during the first year of the pandemic \cite{konstantinoudis2022regional, konstantinoudis2023workflow} . Ensemble hierarchical bayesian spatiotemporal models have been used to quantify the mortality toll of climate extremes such as heatwaves in Switzerland during summer 2022 \cite{konstantinoudis2025ensemble} and tropical cyclones in the US \cite{parks2023short}. Overall, this methodology can be a valuable tool for monitoring a wide range of extremes in the climate change era, including droughts, wildfires, and infectious disease outbreaks.

Other Comments

1. Data Description

The authors should provide summary statistics at the NUTS2 region level, including geographic size, population distribution, and observed mortality rates. Understanding these metrics may help clarify the heterogeneity in blackout effects.

Authors' reply: We have now added a table of summary statistics at the NUTS2 region level in the online supplement, see Table S1 and line 66, page 3:

and region (NUTS2 level; see Online Supplement Figures S1–S2).

Portugal			
NUTSII	Number of deaths	Population	Area
Alentejo	114677	80679	27239
Algarve	79692	76671	4974
Área Metropolitana de Lisboa	436771	476217	2844
Centro	321857	277847	23233
Norte	539302	604193	21256
Oeste e Vale do Tejo	164835	140194	9197
Spain			
Andalucía	1102867	1406803	87577
Aragón	192333	221885	47660
Cantabria	71813	97680	5325
Castilla y León	345937	406913	94076
Castilla-La Mancha	292272	344555	79389
Cataluña	1011217	1270545	32073
Comunidad Foral de Navarra	86910	108541	10385
Comunidad Valenciana	691696	840174	23277
Comunidad de Madrid	642142	1097945	8010
Extremadura	160035	179527	41604
Galicia	487875	453586	29558
Illes Balears	129583	191422	5010
La Rioja	37567	53047	5045
País Vasco	323865	365708	7202
Principado de Asturias	202589	172422	10597
Región de Murcia	172332	249153	11310

2. Results

Authors summarize their main findings in Figure 1 and Table 1 of the paper. Excess mortality is reported only for Spain (lag 0-2 days). Similar effects are not observed across the board which the authors need to explain. In particular,

a. Spain:
    i. Lag 0 days: +14 deaths (95% CrI –59 to +80), +29 females, –15 males — all not statistically significant.
    ii. Lag 0-2 days: 167 (95% CrI +28 to +300), mainly from >84 years group (+99), especially females (+98).
    iii. Lag 0-6 days: –72 (95% CrI –202 to +319), inconclusive; still mainly driven by >84 females.

The spike observed in lag 0-2 days disappears. If the blackout had caused mortality to spike, we might expect elevated cumulative deaths over the full week — unless vulnerable individuals died earlier than they would have, leading to fewer deaths later in the week. But this needs to be explained.

Authors' reply: We thank the reviewer for this comment. We have now added a paragraph on the discussion explaining this, page 6, lines 166-173:

The excess mortality peak observed at lag 0–2 days in Spain, particularly among women aged >84 years, is no longer apparent when we consider the entire week after the blackout. A plausible explanation is a harvesting (or mortality displacement) mechanism, whereby very frail individuals die slightly earlier than they would have in the absence of the blackout. Under this mechanism, an early spike in deaths can be followed by a period (or a day, Figure~\ref{fig:1}) of lower-than-expected mortality, so that cumulative excess mortality over a longer window (lag 0–6 days) is attenuated or even returns towards the null. In addition, estimates over longer lags inherently include more days that were only weakly, if at all, affected by the blackout, which introduces statistical noise and dilutes the signal from the short, intense exposure period.

iv. Sex differences

The authors should explain the peculiarly high mortality among females compared to males in Spain. This contrasts with Portugal, where estimates for females are -20, -23, and -52, and males are -12, +28, +34 for the lag 0, lag0-2, and lag0-6 days, respectively.

Authors reply: We have revised the text as follows, lines 147-149 on pages 5-6:

The absence of comparable effects in older males could be partly explained by the underlying age structure, as women are disproportionately represented in the oldest age groups (Online Supplement Figure S8), where frailty and dependence on electrically powered services are more common \cite{ilincaGenderDifferencesAccess2022}.

We highlight that a subsequent study focusing only on Spain reported similar results, lines 137-142 on page 5:

Our results are consistent with a 2025 blackout study in Spain \cite{garcia2025exploring}. The estimated excess deaths among women at lag 0–2 are compatible across studies, with that paper reporting 125 excess deaths (95% CI -3, +254) and our analysis yielding 139 excess deaths (95% CrI +39, +228). In contrast to their findings, which suggested the largest effects in the 65–84 years age group, we identified people aged >85 years as the most affected. However, the respective uncertainty intervals overlap, indicating that these apparent differences by age group should be interpreted cautiously.

In Portugal there was limited evidence for an effect, apart from younger males for lag0-2. We have now further elaborated, page 6, lines 149-151:

We did not find comparable effects in Portugal, which among other things, could reflect differences in blackout duration, baseline frailty and organisational differences in elderly care.

And on page 6, lines 153-155:

We also found weak evidence of excess mortality among working-age males in Portugal that could reflect random variation or a modest rise in accidental deaths associated with the blackout. Additional research with cause-of-death data and high-resolution exposure metrics is needed to elucidate these mechanisms.

b. Portugal
   i. Lag 0 days: –32 (95% CrI –71 to +4), not statistically significant. Though statistically significant reduced mortality is observed for >84. (–28; 95% CrI –54 to –4), which is counterintuitive given the vulnerability of the age group.
   ii. Lag 0-2 days: +6 (95% CrI –65 to +73), inconclusive. Though a slight increase for 0-64 years (+21; 95% CrI -1, +41 and a slight decrease for >84 years (-17; 95%CrI -62, +27).
   iii. Lag 0-6 days: –19 (95% CrI –154 to +94), inconclusive.

Mortality patterns are inconsistent across countries, sexes, age groups, and lag periods. In the absence of careful explanation, the results could reflect random fluctuations. Addressing point 3 below could help partially explain these patterns.

Authors' reply: We thank the reviewer for this concise summary of the results.

Regarding regional differences, as we also expand in point 3, any more concrete regional comparison would require adjusting for the local duration of the blackout, which varied across regions, and no official record on this exists. We have now added a paragraph about this on page 6, lines 156-165:

Some of the subregional differences could be explained by variation in blackout duration. The blackout began at 12:33 CEST on 28 April 2025 and led to a near-total loss of supply in mainland Spain and Portugal. System-level reports indicate that electricity supply in mainland Spain was largely restored during the evening of 28 April, with approximately 99\%

of demand recovered by 07:00 CEST and full restoration by 11:00 CEST on 29 April. According to the Spanish transmission system operator, parts of northern Spain and Andalusia were reconnected earlier, around 17:00 CEST on 28 April, whereas other areas of the peninsula remained without power for longer. In mainland Portugal, around half of the population had power back by late evening on 28 April and full restoration was achieved in the early hours of 29 April. However, detailed restoration times are not available at sub-national level, which prevents a direct assessment of whether within-country differences in blackout duration account for the observed regional heterogeneity in health impacts.

Regarding age and sex differences we make some speculations, but the lack of more granular data limits the interpretability of the differences, lines 147-149 on pages 5-6:

The absence of comparable effects in older males could be partly explained by the underlying age structure, as women are disproportionately represented in the oldest age groups (Online Supplement Figure S8), where frailty and dependence on electrically powered services are more common \cite{ilincaGenderDifferencesAccess2022}.

We have also added random fluctuations as a potential explanation in some of the patterns observed on page 6, lines 153-155:

We also found weak evidence of excess mortality among working-age males in Portugal that could reflect random variation or a modest rise in accidental deaths associated with the blackout. Additional research with cause-of-death data and high-resolution exposure metrics is needed to elucidate these mechanisms.

Regarding discrepancies between lag periods we have now provided a paragraph in the discussion on page 6, lines 166-173:

The excess mortality peak observed at lag 0–2 days in Spain, particularly among women aged >84 years, is no longer apparent when we consider the entire week after the blackout. A plausible explanation is a harvesting (or mortality displacement) mechanism, whereby very

frail individuals die slightly earlier than they would have in the absence of the blackout. Under this mechanism, an early spike in deaths can be followed by a period (or a day, Figure~\ref{fig:1}) of lower-than-expected mortality, so that cumulative excess mortality over a longer window (lag 0–6 days) is attenuated or even returns towards the null. In addition, estimates over longer lags inherently include more days that were only weakly, if at all, affected by the blackout, which introduces statistical noise and dilutes the signal from the short, intense exposure period.

3. Data Limitations and Confounding Concerns

   a. No cause-of-death data utilized, which could be helpful in linking blackout effects to mortality (e.g., vulnerable individuals requiring constant care via electric medical equipment).
   b. The death records used by the authors lack the exact date of death and may include reporting delays, leading to possible misclassification of day-of-event deaths. Even a one-day delay could mean that deaths recorded on the day of the blackout occurred the previous day. The authors could partially address points 3a and 3b by triangulating their results using a subsample of records with precise cause and day of death (for example, via data from regional hospitals).

Authors reply: We do not have access to cause-specific mortality, only all-cause mortality by date of death. We already acknowledge that cause-of-death data would be valuable to disentangle these pathways and that our estimates therefore capture the aggregate mortality impact of the blackout rather than specific causes. Regarding reporting delays, while they can affect when deaths are registered in administrative systems, they do not affect the recorded date of death, which is assigned at the time of death and used consistently in the datasets analysed. As a result, delays in notification should not systematically shift deaths from the day before the blackout to the blackout day itself.

In lines 94–96, the authors attribute >84 female excess mortality in Spain to the vulnerability of certain care settings (outpatient medical services, domiciliary nursing, and care homes). However, similar effects for >84 males are absent (+2; 95% CrI -56, +60).

Authors reply: We have now elaborated on sex differences, lines 147-149 on pages 5-6:

The absence of comparable effects in older males could be partly explained by the underlying age structure, as women are disproportionately represented in the oldest age groups (Online Supplement Figure S8), where frailty and dependence on electrically powered services are more common \cite{ilincaGenderDifferencesAccess2022}.

And added a plot on the supplement showing the differences in the age distribution between males and females:

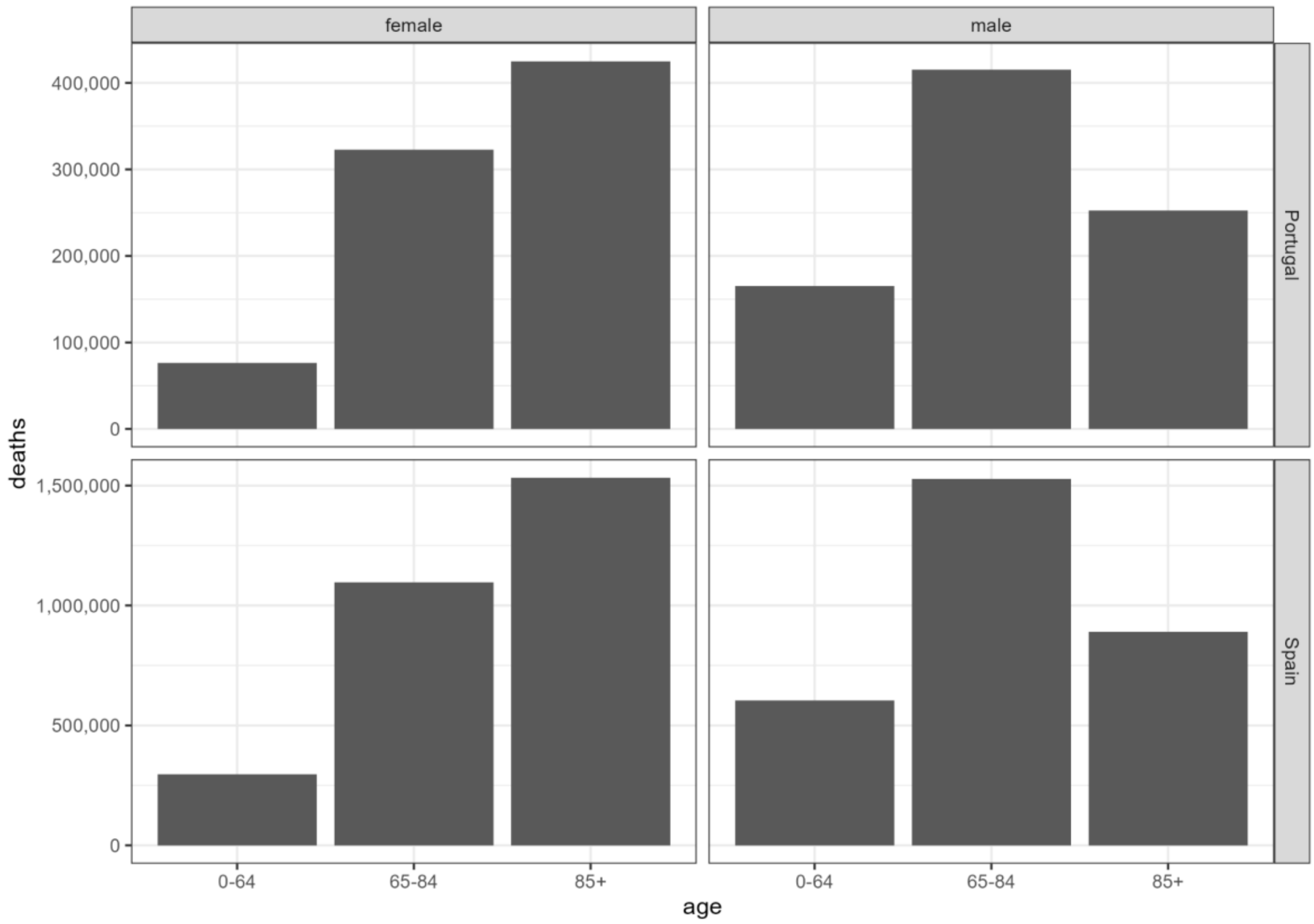

c. In lines 96-98, the authors cite that differences in the health care systems of Spain and Portugal are a partial explanation for the increased mortality in Spain. The authors indicate that the Spanish health care system is marked by regional

disparities. Is there a documented difference in the performance of these healthcare systems across regions, and does that correlate to the authors' findings of mortality rates in different regions in Spain?

Authors reply: We agree that a further analysis of regional differences would be relevant. However, we are unable to perform such an analysis at this stage. Any regional comparison would require adjusting for the local duration of the blackout, which varied across regions, and no official record on this exists, see page 6, lines 156-165:

Some of the regional differences could be explained by variation in blackout duration. The blackout began at 12:33 CEST on 28 April 2025 and led to a near-total loss of supply in mainland Spain and Portugal. System-level reports indicate that electricity supply in mainland Spain was largely restored during the evening of 28 April, with approximately 99\% of demand recovered by 07:00 CEST and full restoration by 11:00 CEST on 29 April. According to the Spanish transmission system operator, parts of northern Spain and Andalusia were reconnected earlier, around 17:00 CEST on 28 April, whereas other areas of the peninsula remained without power for longer. In mainland Portugal, around half of the population had power back by late evening on 28 April and full restoration was achieved in the early hours of 29 April. However, detailed restoration times are not available at sub-national level, which prevents a direct assessment of whether within-country differences in blackout duration account for the observed regional heterogeneity in health impacts.

d. In lines 100-101, the authors “found weak evidence of excess mortality among working-age males in Portugal, likely reflecting a modest rise in accidental deaths associated with the blackout.” A similar effect is not observed for working-age males in Spain. Why did a similar increase not happen in Spain?

Authors’ reply: We agree with the reviewer that this difference is not something we can explain with our data, so we decided to tone this down and adapted the text as follows (page 6, lines 153-155):

We also found weak evidence of excess mortality among working-age males in Portugal that could reflect random variation or a modest rise in accidental deaths associated with the blackout. Additional research with cause-of-death data and high-resolution exposure metrics is needed to elucidate these mechanisms.

4. Presentation

   a. Line 122, replace ‘out’ with ‘our’.

Authors’ reply: We implemented accordingly.

   b. A visual representation of Table 1 would help grasp the findings more conveniently. See the example below (may not accurately represent all the mortality numbers).

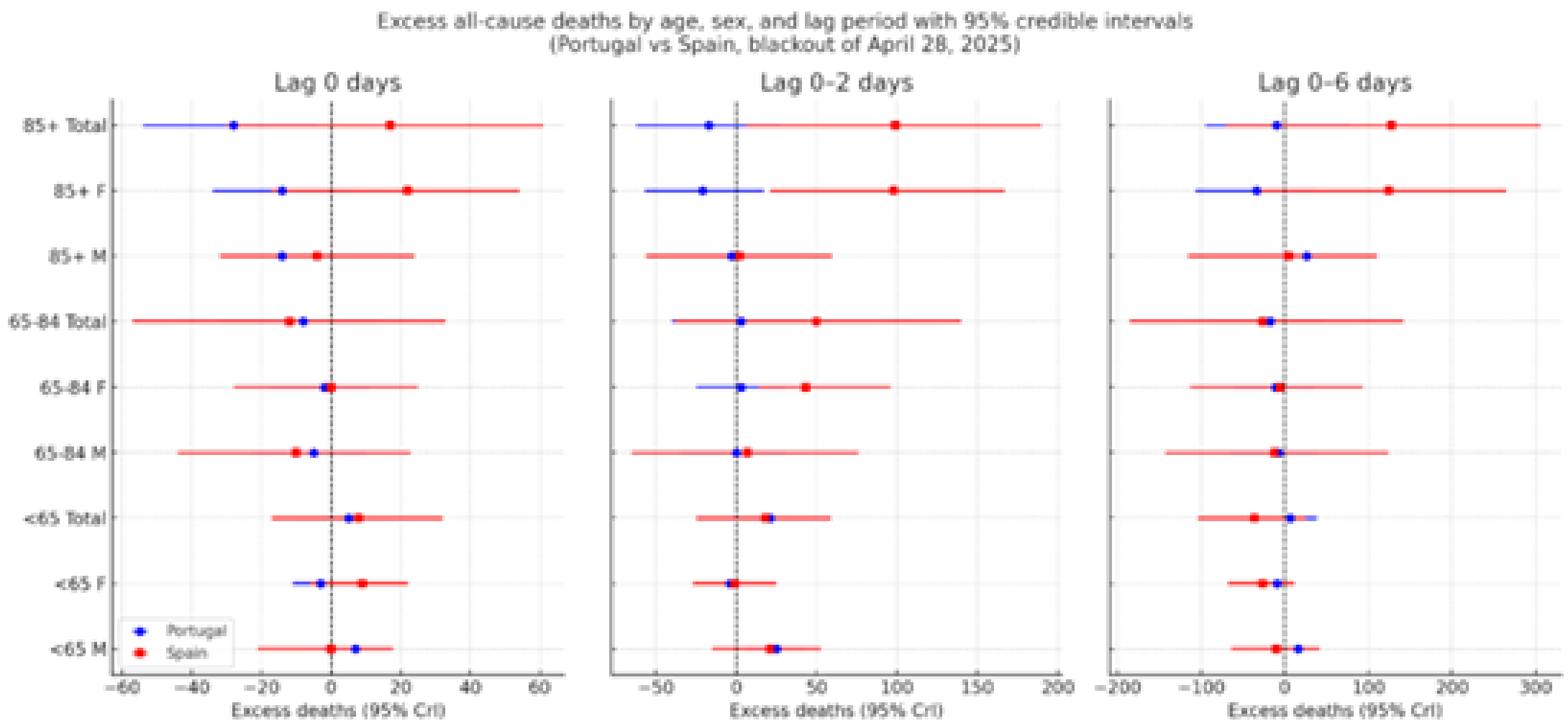

Authors’ reply: We thank the reviewer very much for this suggestion. This plot is very nice and conveys the message very clearly. We have now included a similar one in the supplement, stratifying by country, see Online Supplement Figure S3 and bellow:

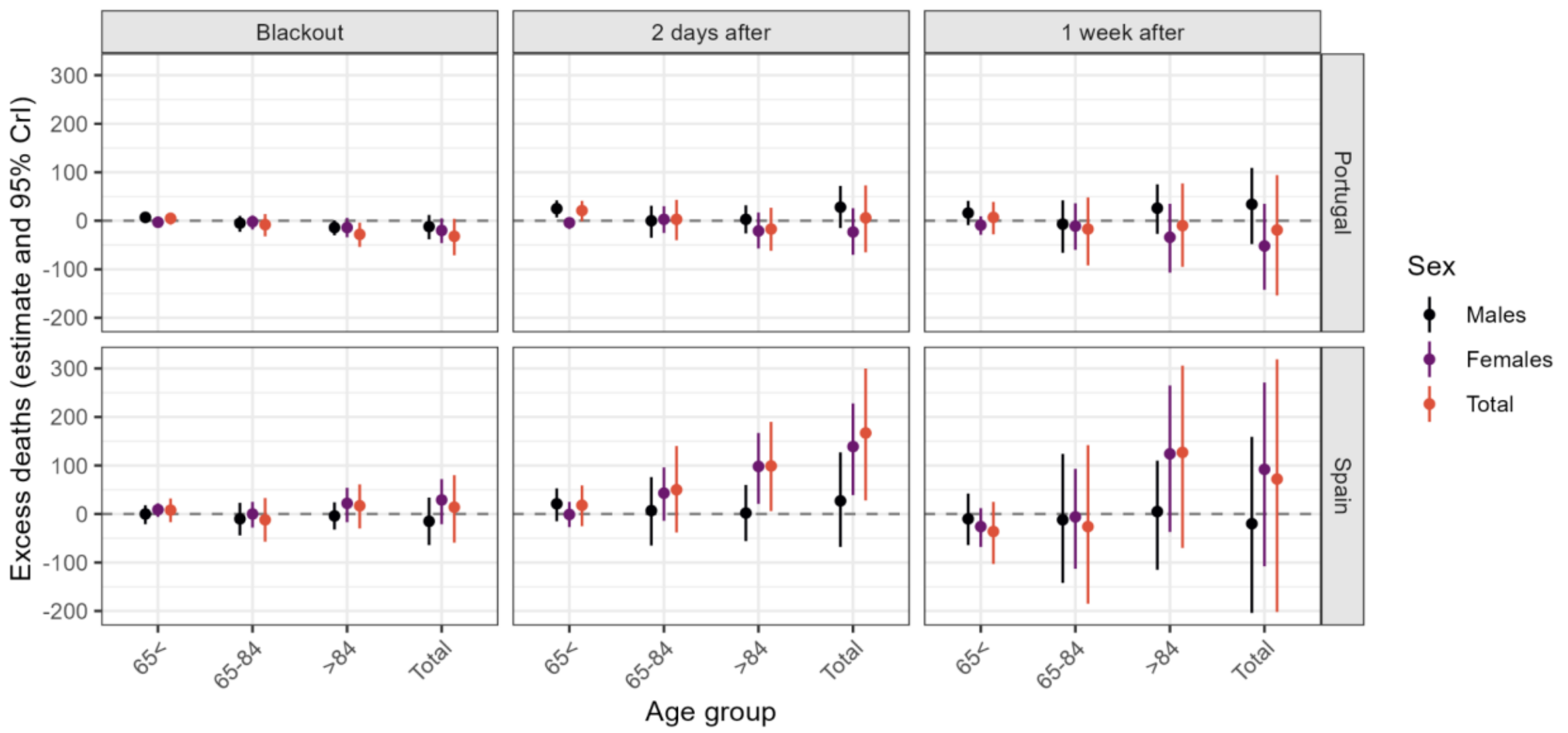

5. Model Specifications The authors should briefly explain why the specific model specifications were used and why M3 performed slightly better than M2 and M1. M3 utilizes a second-order random walk, which provides a smooth data-driven curve that avoids overfitting to small random noise. This is appropriate for mortality trends that change gradually without erratic week-to-week jumps.

Authors' reply: We have now added a couple of sentences in the methods section (page 9, lines 249-252):

We modelled δ_{*} using three specifications with increasing complexity, allowing us to examine a range of different temporal trends (random noise, linear, and non-linear). Model 1 assigns an unstructured random effect on calendar time to model temporal trends, model 2 adds a linear trend in calendar time on model 1 and model 3 models calendar time using a structured temporal random effect:

Model 3 performs best as for most age and sex groups the long-term trends are non-linear. We decided not to include this sentence in the manuscript as this would have required defining the models in the result section, which comes before the methods in nature communications.

Please also note that we have now separated results from the discussion being in line with the format in nature communications.

Reviewer 2

I co-reviewed this manuscript with one of the reviewers who provided the listed reports. This is part of the Nature Communications initiative to facilitate training in peer review and to provide appropriate recognition for Early Career Researchers who co-review manuscripts.

Author's reply: Thank you, see our comments above.

Reviewer 3

This manuscript focuses on the recent Iberian Blackout of 2025, which impacted broad populations and lasted up to ten hours. The authors leverage publicly available all-cause mortality data and use Bayesian time series methods to estimate the impact of the blackout on the same-day as the event, lags 0-2, and over the following week. Differences in impact by country, region, age, and sex emerge. The manuscript is well written and appropriately characterizes the findings. I have a few suggestions to improve the work.

Major

1. Lines 93-101: while I agree with some of the speculation from the authors as to why sex and age and county-specific differences were observed, this section seems too speculative for a scientific manuscript. Please present alternative reasons and cite sources for the claims made in the paragraph.

Authors' reply: We revised this paragraph, decreasing the speculations and adding several supporting references (pages 5-6, lines 143-155):

Our reported estimates of excess mortality varied across countries, regions or demographic groups, suggesting that the impact of the blackout was variable across contexts and settings. In Spain, the concentration of excess mortality in females aged 85 or older is consistent with disruptions affecting outpatient medical services, domiciliary nursing and care homes, settings in which power outages have been linked with increased short-term mortality \cite{skarhaAssociationPowerOutage2021}. The absence of comparable effects in older males could be partly explained by the underlying age structure, as women are disproportionately represented in the oldest age groups (Online Supplement Figure S8), where frailty and dependence on electrically powered services are more common \cite{ilincaGenderDifferencesAccess2022}. We did not find comparable effects in Portugal, which among other things, could reflect differences in blackout duration, baseline frailty and organisational differences in elderly care. Indeed, Portugal has a highly centralised and

integrated public long-term care system \cite{fonsecaLongtermCareUnits2023}, whereas the Spanish system is characterized by decentralisation and marked territorial disparities \cite{diaz-tenderoLongTermCarePolicies2024}. We also found weak evidence of excess mortality among working-age males in Portugal that could reflect random variation or a modest rise in accidental deaths associated with the blackout. Additional research with cause-of-death data and high-resolution exposure metrics is needed to elucidate these mechanisms.

2. Modeling approach: Should any inclusion of circulating respiratory viruses be considered that might impact mortality?

Authors' reply: The reviewer is right in pointing out that respiratory virus circulation can affect short-term mortality, so we checked routine surveillance for April 2025. There is no indication of an unusual influenza or COVID-19 wave in either country during that period. We have now added the following sentence in the discussion (page 7, lines 200-203):

Although respiratory virus circulation can influence mortality, surveillance data indicate no unusual influenza or COVID-19 activity in Spain or Portugal during April 2025, making concurrent epidemic peaks an unlikely explanation for the observed patterns.

3. Signals are detected and modeling performance is good, but I would emphasize the range of the credible interval further (from 28 to 300).

Authors' reply: We have now added a sentence acknowledging the range of the credible interval (page 3, lines 81-83):

The 95\% credible interval of +28 to +300 excess deaths reflects the range of effects that remain reasonably compatible with the data and model, acknowledging substantial uncertainty in the exact magnitude of the excess mortality.

4. Given the observed spatial heterogeneity is there any further blackout data available to

link duration of the event across space? I assume that the blackout did not last the same duration across the study region and this might help explain observed differences.

Authors' reply: Thank you for the important comment. We have now added a paragraph on page 6, lines 156-165:

Some of the regional differences could be explained by variation in blackout duration. The blackout began at 12:33 CEST on 28 April 2025 and led to a near-total loss of supply in mainland Spain and Portugal. System-level reports indicate that electricity supply in mainland Spain was largely restored during the evening of 28 April, with approximately 99\% of demand recovered by 07:00 CEST and full restoration by 11:00 CEST on 29 April. According to the Spanish transmission system operator, parts of northern Spain and Andalusia were reconnected earlier, around 17:00 CEST on 28 April, whereas other areas of the peninsula remained without power for longer. In mainland Portugal, around half of the population had power back by late evening on 28 April and full restoration was achieved in the early hours of 29 April. However, detailed restoration times are not available at sub-national level, which prevents a direct assessment of whether within-country differences in blackout duration account for the observed regional heterogeneity in health impacts.

Minor

1. Abstract mentions direct effect of power outage is fire. This link is not immediately clear and should be clarified or removed from the abstract.

Authors' reply: We agree with the reviewer and removed the mention of "fire" from the abstract.

2. Lines 30-31: please explain how CO poisoning occurs, this is tied to improper generator use. Please describe how fires occur, is this tied to candle use?

Authors' reply: The mechanisms linking outages, CO poisoning and fire were only briefly mentioned in the second paragraph, which we adapted as follows (page 2, lines 30-33):

Power outages can affect health through a range of mechanisms. Injuries and fatalities can occur when households use alternative light, heat or power sources during outages, which can result in carbon monoxide poisoning from fuel-burning heaters or generators in enclosed or poorly ventilated spaces, and fires associated with unattended candles or improvised indoor heating and cooking practices \cite{Casey2020, klingerPowerOutagesExtreme2014}.

3. Line 63: Did excess deaths over the week following the fire become inconclusive or evidence was found of no excess mortality in the week following the blackout?

Authors' reply: Thank you for this comment, we have now reformulated:

However, we found no excess mortality in the week following the blackout.

4. Figure 1: why are the credible intervals multiple shades of blue?

Authors' reply: Thank you for this comment, the different shades of blue represent different percentiles, i.e. 2.5%, 5%, 10%, 20%, 30%, 70%, 80%, 90%, 95% and 97.5%. We have now added a sentence on the legend justifying this.

The different shades of blue represent different percentiles of the posterior distribution (2.5\%, 5\%, 10\%, 20\%, 30\%, 70\%, 80\%, 90\%, 95\% and 97.5\%).

5. Figure 2 presenting geographic heterogeneity on relative scale and probability of excess is useful

Authors' reply: Thank you.

6. Supplemental Figure 1: please describe what models 1, 2, and 3 are in the figure caption.

Authors' reply: We have now defined the 3 different models in the caption for Supplemental Figures 1 and 2:

All models include an intercept, the effect of public holidays, a non-linear and lagged effect of temperature, a spatial autocorrelation term, a day of the week effect and a seasonality term. Model 1 (M1) uses an unstructured random effect on calendar time to model temporal

trends, Model 2 (M2) adds a linear trend in calendar time on model 1 and Model 3 (M3) models calendar time using a structured random effect.

7. Line 84: a brief description of differences in the regions of Spain would be helpful. What is different about Northern Spain, for example?

Authors' reply: We agree that a further analysis of regional differences would be relevant. Addressing the comments by reviewers 4 and 5 we have now run a sensitivity analysis assessing how *GDP* explains regional differences.

First we provide a plot of the GDP in the Online supplement, see Figure S6.

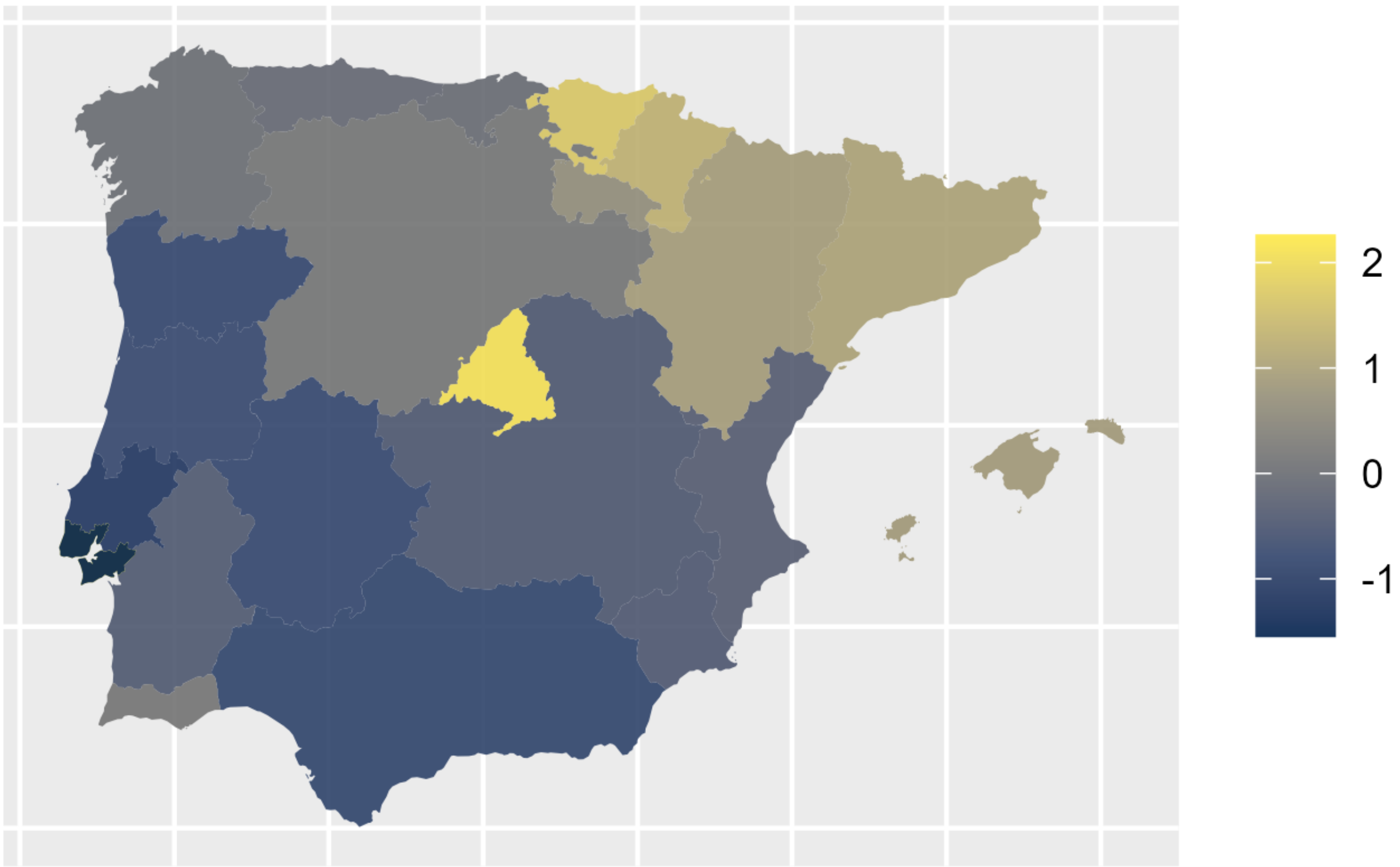

Then on pages 4-5, lines 113-118 we provide the results of the sensitivity analysis:

We last investigated the association between regional excess mortality and socioeconomic context, as proxied by gross domestic product (GDP) per capita. NUTS2-level GDP per capita for Spain and Portugal was obtained from Eurostat in 2023 and linked to the corresponding regional estimates of excess mortality. Visual inspection of scatterplots and correlation patterns across the different lags indicated no clear evidence of a systematic

relationship between GDP per capita and the magnitude of excess mortality, Online Supplement Figure S6-7.

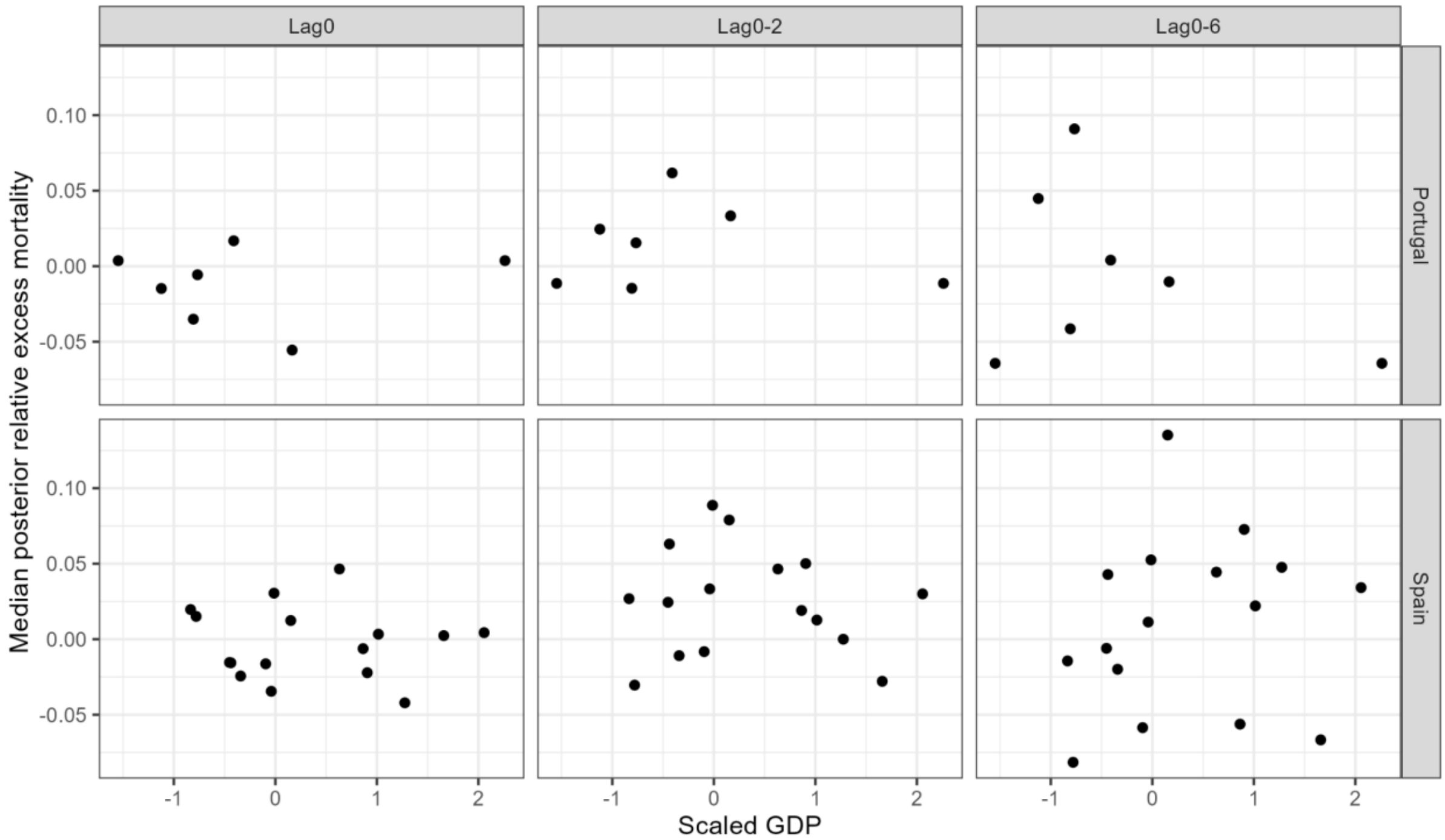

And in methods (pages 10-11, lines 296-302):

Finally, we examined the association between regional excess mortality and socioeconomic context, proxied by gross domestic product (GDP) per capita. NUTS2-level GDP per capita data for Spain and Portugal were obtained from Eurostat (2023) and linked to the corresponding regional estimates of excess mortality. We then plotted the median posterior excess mortality alongside scaled GDP per capita for Spain and Portugal to assess whether regional differences could be explained by variations in GDP.

8. Line 153-159: have others used a similar approach to handle the COVID-19 pandemic effects? Please provide citations if so.

Authors' reply: Thank you for the comment. We do not know any other study that accounted for the COVID-19 pandemic in a similar way. Most studies remove the COVID-19 years from the analysis. We have now added a recent study focusing only on the blackout in Spain that removed the pandemic year (page 2, lines 45-55):

A recent study examining the 2025 blackout, focusing on Spain, instead used historical data and a generalised additive model to predict the mortality that would have occurred in the absence of the blackout, thereby adopting a counterfactual framework \cite{garcia2025exploring}. This approach has several appealing features: the number of deaths can, in principle, be validated through cross-validation, and the method does not rely on specific assumptions about the exposure–response relationship. This counterfactual approach has several advantages: the number of deaths can be validated, through cross-validation, and the method does not rely on assumptions related to the exposure–response relationship. However, that application did not report model validation, did not account for spatial autocorrelation and excluded the COVID-19 pandemic period from the training set \cite{garcia2025exploring}. Ignoring spatial correlation can lead to underestimated uncertainty, while removing the COVID-19 years, rather than explicitly modelling their effects, may fail to capture recent mortality trends and variability.

Reviewer 4

First of all, I would like to thank the authors for the importance of this study and its innovative methodological implementation. It is a very well-structured and explained piece of work.

The only thing I found lacking in the analysis was the income factor. In Spain, there are rural areas, even provinces, where the population has very, very low incomes, and this can make death due to this type of event more likely.

One suggestion, if possible, would be to include the income or GDP of the provinces, which would be interesting (if possible).

Thank you very much for such an interesting topic.

Authors' comment: We thank the reviewer for the suggestion. We have now downloaded GDP per NUTSII region as retrieved from EUROSTAT.

First we provide a plot of the GDP in the Online supplement, see Figure S6.

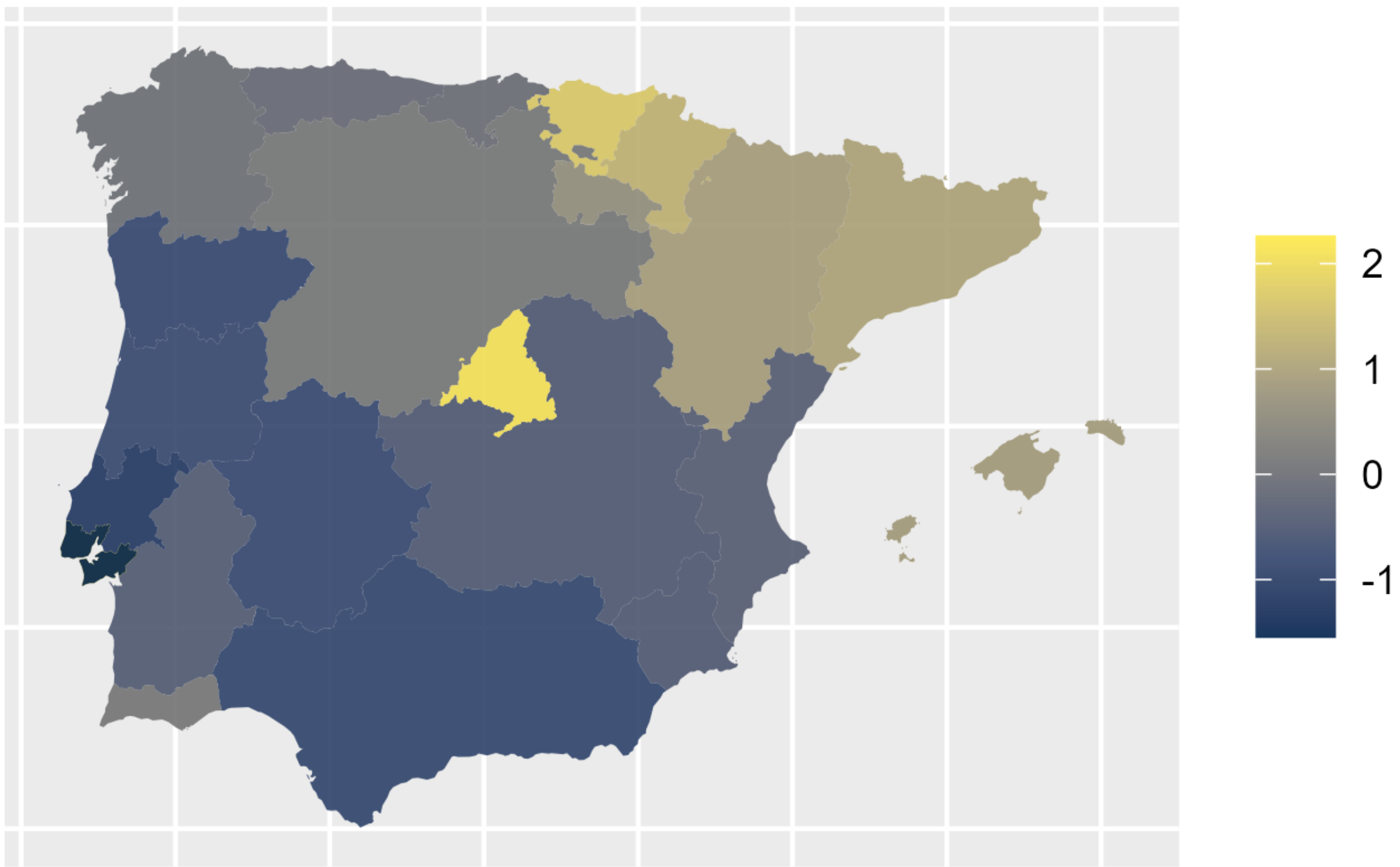

Then on pages 4-5, lines 113-118 we provide the results of the sensitivity analysis:

We last investigated the association between regional excess mortality and socioeconomic context, as proxied by gross domestic product (GDP) per capita. NUTS2-level GDP per capita for Spain and Portugal was obtained from Eurostat in 2023 and linked to the corresponding regional estimates of excess mortality. Visual inspection of scatterplots and correlation patterns across the different lags indicated no clear evidence of a systematic relationship between GDP per capita and the magnitude of excess mortality, Online Supplement Figure S6-7.

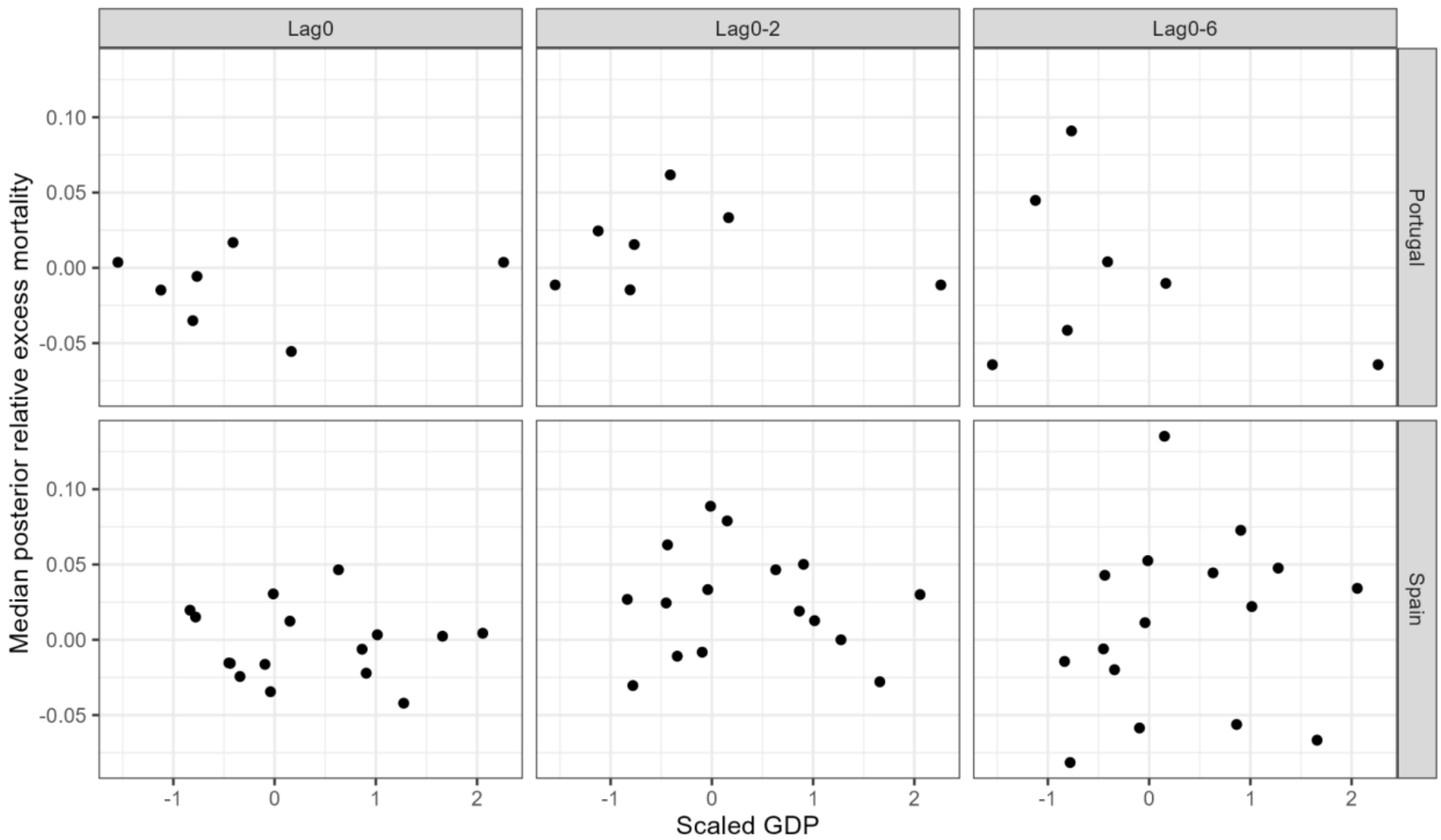

And in methods (pages 10-11, lines 296-302):

Finally, we examined the association between regional excess mortality and socioeconomic context, proxied by gross domestic product (GDP) per capita. NUTS2-level GDP per capita data for Spain and Portugal were obtained from Eurostat (2023) and linked to the corresponding regional estimates of excess mortality. We then plotted the median posterior excess mortality alongside scaled GDP per capita for Spain and Portugal to assess whether regional differences could be explained by variations in GDP.

Reviewer 5

In this manuscript, the authors present the first systematic assessment of all-cause excess mortality associated with the April 2025 blackout that affected mainland Portugal and Spain. Using daily mortality and population data at the NUTS2 level, stratified by age and sex, the authors fit Bayesian spatiotemporal models to estimate counterfactual expected deaths for the blackout week. The modelling framework incorporates distributed lag non-linear temperature effects, long-term and seasonal trends, day-of-week patterns, holidays, and regional random effects, and is implemented using INLA. Excess mortality is then quantified by comparing observed deaths with the model-based predictions across several predefined lag windows. The results suggest no clear excess mortality on the day of the blackout, but indicate a short-term increase in Spain over the subsequent two days, particularly among women aged 85+. No consistent signal is found in Portugal. The authors interpret these patterns as evidence of heterogeneous vulnerability to blackout-related disruptions, with possible implications for healthcare resilience. The topic is timely and relevant, and the modelling effort is substantial. The work has potential value for understanding health impacts of large-scale infrastructural failures. However, several aspects of the study design, model specification, and interpretation would benefit from further clarification and refinement before the conclusions can be fully supported.

The paper

Major comments

1. Air pollution as an omitted confounder (lines 163 -)

The models adjust for temperature using a DLNM framework, but do not include any air-pollution covariates. Given the well-established short-term effects of PM2.5, PM10 and NO2 on daily mortality, and the fact that blackouts may temporarily alter emissions through generator use, traffic disruption or indoor combustion, a clarification would be welcome.

Even if pollution data were unavailable or too coarse, briefly discussing the potential for confounding, or providing a sensitivity assessment, would help strengthen the interpretation of the excess mortality estimates.

Author's reply: We thank the reviewer for these important comments. We have now added a couple of sentences justifying why we did not include air-pollution in the limitations (page 7, lines 197-201):

We did not incorporate air pollution in our predictive model. Including air pollution as a covariate in the prediction model would be expected to block part of the blackout effect operating through changes in air pollution. If blackout events modify emissions and therefore air-pollution levels, air pollution would lie on the causal pathway between blackout and mortality; conditioning on such a mediator would potentially bias the estimated total effect of the blackout.

2. Socioeconomic determinants and baseline vulnerability

Given the clear heterogeneity in excess mortality by region and by age–sex group, it would be helpful to discuss more explicitly the role of underlying socioeconomic conditions.

Author's reply: We have now downloaded GDP per NUTSII region as retrieved from First we provide a plot of the GDP in the Online supplement, see Figure S6.

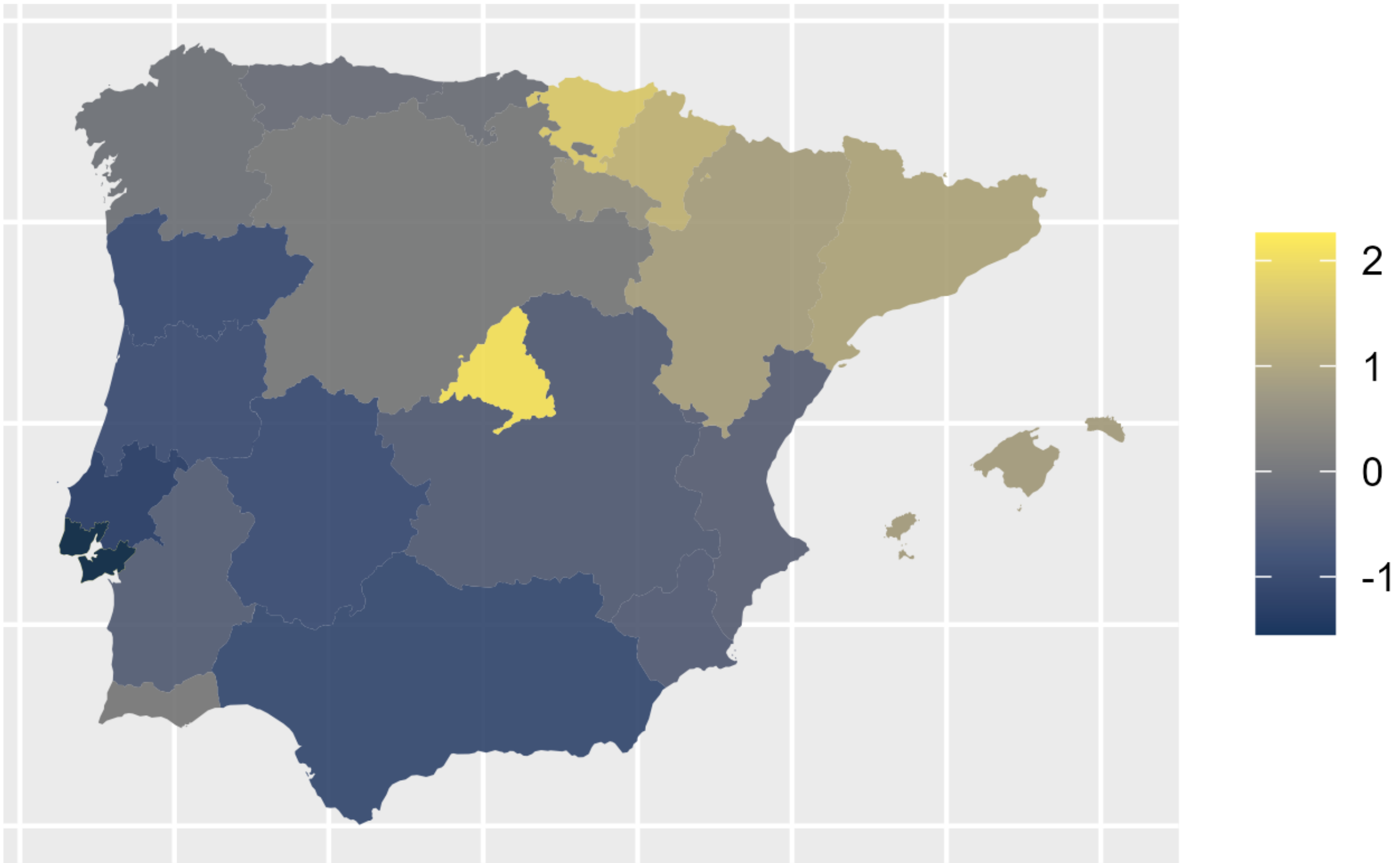

Then on pages 4-5, lines 113-118 we provide the results of the sensitivity analysis:

We last investigated the association between regional excess mortality and socioeconomic context, as proxied by gross domestic product (GDP) per capita. NUTS2-level GDP per capita for Spain and Portugal was obtained from Eurostat in 2023 and linked to the corresponding regional estimates of excess mortality. Visual inspection of scatterplots and correlation patterns across the different lags indicated no clear evidence of a systematic relationship between GDP per capita and the magnitude of excess mortality, Online Supplement Figure S6-7.

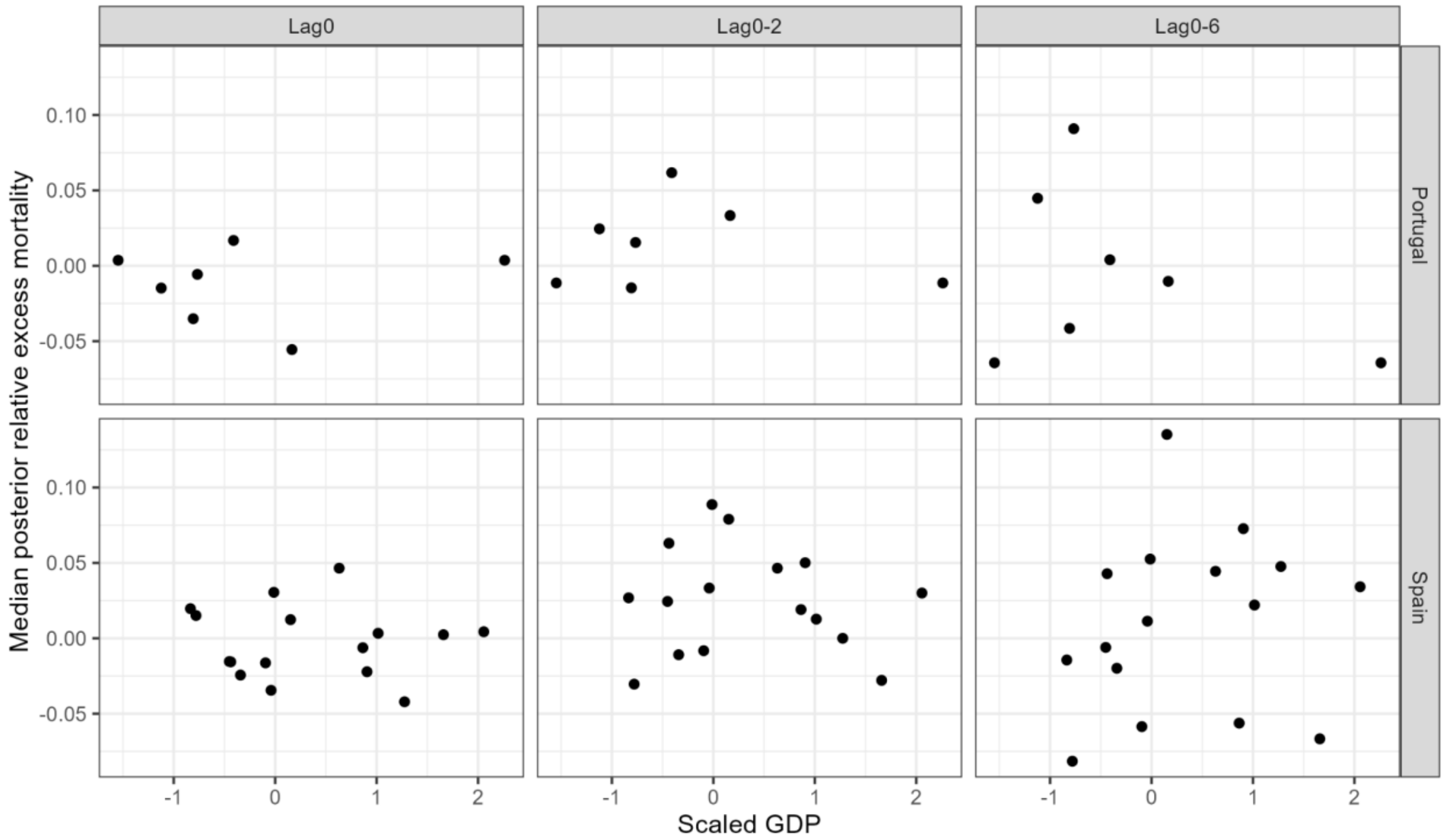

And in methods (pages 10-11, lines 296-302):

Finally, we examined the association between regional excess mortality and socioeconomic context, proxied by gross domestic product (GDP) per capita. NUTS2-level GDP per capita data for Spain and Portugal were obtained from Eurostat (2023) and linked to the corresponding regional estimates of excess mortality. We then plotted the median posterior excess mortality alongside scaled GDP per capita for Spain and Portugal to assess whether regional differences could be explained by variations in GDP.

3. Clarification of national holidays included in the model (line 164) The manuscript mentions the inclusion of national holidays as a binary covariate, but it is not clear which specific holidays were incorporated for each country and whether regional holidays were also considered. This detail matters because holiday calendars could differ across autonomous communities in Spain, and in 2025, 28 April was a public holiday in the Valencian Community. It would be helpful if the authors could clarify.

Author's reply: We thank the reviewer for this comment. We have downloaded public holidays from date.nager.at and incorporated regional holidays at the NUTSII level. We have now clarified in the text (page 9, line 246):

public holidays (accounting for regional holidays as well)

4. Specification of the covariate $X2,mt$ in Model 2 (line 170)

In Model 2, $X2,mt$ is described as "the week and the year", but this definition is ambiguous. It would be helpful if the authors could specify whether $X2,mt$ represents a linear trend in calendar time, a combination of week number and year indicator, or some other coding scheme. Because this term interacts with seasonality and long-term trends, a clearer description would assist readers in understanding how Model 2 differs structurally from Models 1 and 3.

Author's reply: Thank you for the comment. We have now added some sentences to make it clearer (page 9, lines 250-253):

We modelled δ_{*} using three specifications with increasing complexity, allowing us to examine a range of different temporal trends (random noise, linear, and non-linear). Model 1 assigns an unstructured random effect on calendar time to model temporal trends, model 2 adds a linear trend in calendar time on model 1 and model 3 models calendar time using a structured temporal random effect:

5. Justification for knot placement in the B-spline basis (line 174)

The DLNM uses quadratic B-splines with internal knots placed at 10°C, 18°C and 27°C, but the manuscript does not discuss why these particular values were selected. While these temperatures may be reasonable in the Iberian context, the modelling of temperature–mortality relationships is sensitive to knot choice. A short justification—empirical,

climatological, or based on prior literature—or a brief sensitivity check would strengthen confidence in the robustness of the temperature adjustment.

Author's reply: We have now added the justification of our selection (page 9, lines 258-261):

For the choice of temperature we specified a quadratic B-spline with 5 degrees of freedom for temperature, with knots placed by default at equally spaced value in the space of the predictor as suggested previously \cite{gasparrini2013distributed}.

And a sensitivity analysis using another well established parametrisation (page 4, lines 110-113):

To assess robustness of the results with respect to the different choices made to model the non-linear and lagged effect of temperature \citep{gasparrini2015mortality}, we tested another common parametrisation used in the literature \cite{masselot2023excess} and found that the results were identical, Online Supplement Figure S5.

And page 10 lines 294-298:

To assess the robustness of our results to alternative temperature parameterisations, we specified a quadratic B-spline with knots placed at the 10th, 75th, and 90th percentiles of the region-specific temperature distribution. For the lag dimension, we employed a cubic natural spline with three knots positioned at equally spaced values on the logarithmic scale, assuming a lag period of 21 days \cite{masselot2023excess}

6. Spatial structure of regional random effects (line 177) The manuscript models the regional random effects zs as i.i.d. normal, arguing that this choice is adequate given the relatively large size of NUTS2 regions. However, neighbouring regions are likely to share important unmeasured determinants of mortality (healthcare organisation, socio-demographic profiles, infrastructure, climate), so some degree of spatial correlation in the residuals would be plausible. I would encourage the authors either to provide more justification for the i.i.d.

assumption (e.g., via residual diagnostics such as Moran’s I) or, if feasible, to report a brief sensitivity analysis using a structured spatial prior (e.g., CAR/BYM2). This would help to assess whether the estimated excess mortality is robust to alternative assumptions about spatial dependence.

Authors' reply: We thank the reviewer for this important comment. We have now added a sensitivity analysis using the BYM2 prior to model space and we report the results in the Online Supplement.

Page 4, lines 107-110:

Our initial choice for modelling space across the 3 model candidates did not account for spatial autocorrelation; a decision driven by the very few regions available in Portugal (6 regions). In the first sensitivity analysis, we relaxed this assumption for the best performing model and modelled space using a reparametrisation of the Besag-York-Mollie model \cite{rieblerbym2}.

And page 10, lines 293-294:

In the first sensitivity analysis, we replaced the unstructured prior (iid) used to model spatial autocorrelation with a reparameterisation of the Besag–York–Mollié model \cite{rieblerbym2}.

7. Clarification of the prior on the intercept (line 179)

The manuscript states that the intercept is assigned a “normal prior with mean zero and zero precision”. Since in INLA this parametrisation corresponds to an improper uniform prior on the real line (i.e., a flat prior implemented via infinite variance), it may be clearer to describe it explicitly as such. I would encourage the authors to replace the current wording with a formulation such as “an improper uniform prior for the intercept”, which more accurately

reflects the prior structure and avoids potential confusion for readers unfamiliar with INLA's internal parametrisation.

Authors' reply: We have now implemented accordingly (page 9, line 265):

an improper uniform prior to the intercept

8. PC priors for random-effect hyperparameters (LINE 180–182)

The description of the penalised complexity (PC) priors is rather brief. Given the number of latent components in the model, each of which introduces its own precision hyperparameter (e.g., the RW2 temporal trend, seasonal effects, and the DLNM temperature basis), it would be helpful for the authors to provide a clearer and more comprehensive account of all hyperparameters involved and the specific PC priors assigned to them. In particular, RW2 terms generate a precision parameter with its own PC prior, while spline-based components may rely on different penalisation structures. If any SPDE-based representation or other sparse formulations were used for the B-splines, the associated PC priors may differ accordingly. A more explicit description of how each hyperparameter is defined and penalised would improve transparency and help readers understand the regularisation applied throughout the model.

Authors' reply: We decided to model the non-linear and prolonged effects of temperature on health using the DLNM approach, which basically combines the basis function across the temperature and lag domain through the construction of a cross-basis matrix. We have added the coefficients of the cross-basis matrix as fixed effects in the model and thus the priors are N(0, 1000). We have added a sentence to clarify this (page 9, lines 266-267):

These also include the coefficients of the crossbasis function, which account for the non-linear and prolonged effects of temperature on health.

We have also added a couple of sentences describing the penalised complexity priors and our choices (pages 9-10, lines 268-272):

PC priors shrink toward a parsimonious base model (typically corresponding to no random effect), with the degree of shrinkage controlled through interpretable probability statements on the variance parameter \citep{pcpriors}. Using such probability statements, we calibrated the priors to ensure it is unlikely that relative risks exceed $\exp(2)$ due solely to spatial or temporal variation.

Minor Comments

- Typographical error in affiliation (line 3) “Evnironment” should be corrected to “Environment”.

Authors' reply: We have now corrected the typo.

- Consistency in spelling: “behavior/behaviour” and “organisation/organization” The manuscript mixes British and American spellings. It may be preferable to standardise this.

Authors' reply: We have now implemented accordingly.

- Unclear reference to “the model” in the Results (lines 64–75) At several points, the text refers simply to “the model” without specifying which of the three candidate models is being discussed. Adding the model number would avoid ambiguity.

Authors' reply: We have clarified accordingly (page 3, line 72):

All results reported in the following sections are based on the best-performing model.

We decided on this specification, as we think it is more in line with the structure of nature communications (results coming before methods).

· Ambiguity in “the week of the blackout” (LINE 49) Specify that this refers to 28 April–4 May 2025 for clarity.

Authors' reply: We have implemented accordingly.

· Typographical issues: duplicated commas and inconsistent spacing (e.g., lines 96–100) A quick proofread would resolve minor spacing inconsistencies, such as double spaces after commas.

Authors' reply: We have implemented accordingly.

· Acronym definition (line 111) DLNM (Distributed Lag Non-linear Model) is used before being spelled out. Define at first occurrence for clarity to non-specialist readers.

Authors' reply: We have implemented accordingly.

· Typographical error (line 122) The text reads “out study lacks detailed data…”, which should be corrected to “our study”.

Authors' reply: We have implemented accordingly.

· Non-functional GitHub link (lines 209–211) The manuscript provides a GitHub repository for reproducing the analysis (https://github.com/jriou/ blackout-burden), but the link returns an error.

Authors' reply: We have implemented accordingly.

Point by point reply

Reviewer 1

Based on my co-review, we feel the authors have largely addressed the comments and suggestions in the first round of review. We have only one additional request of the authors. We do not need to review the next revision, if the editors consider the revision responsive. We had asked for region-level details such as mortality rates that may explain the heterogeneity in the mortality seen across the different regions, as provided in Figure 2 in the main paper. However, the table added to the paper provides little information, or the information is hard to match because the regions in the table do not clearly map to the regions in the figure. Two ways to make the connection cleaner would be to either include a numerical table of the results at the region level or to provide a heatmap of relevant region-level statistics in a similar format. Moreover, the table provides one data point per region for the period 2010–25. Either a year-by-year breakdown or a single data point covering only the most recent few years would be a better gauge.

Authors' reply: We thank the reviewers for their follow-up comment. Following their suggestion, we have included annual maps of mortality rates per 1,000 population for NUTS2 regions. We did not include 2025, as it only covers the first 4 months, making the results not directly comparable with those for complete calendar years.

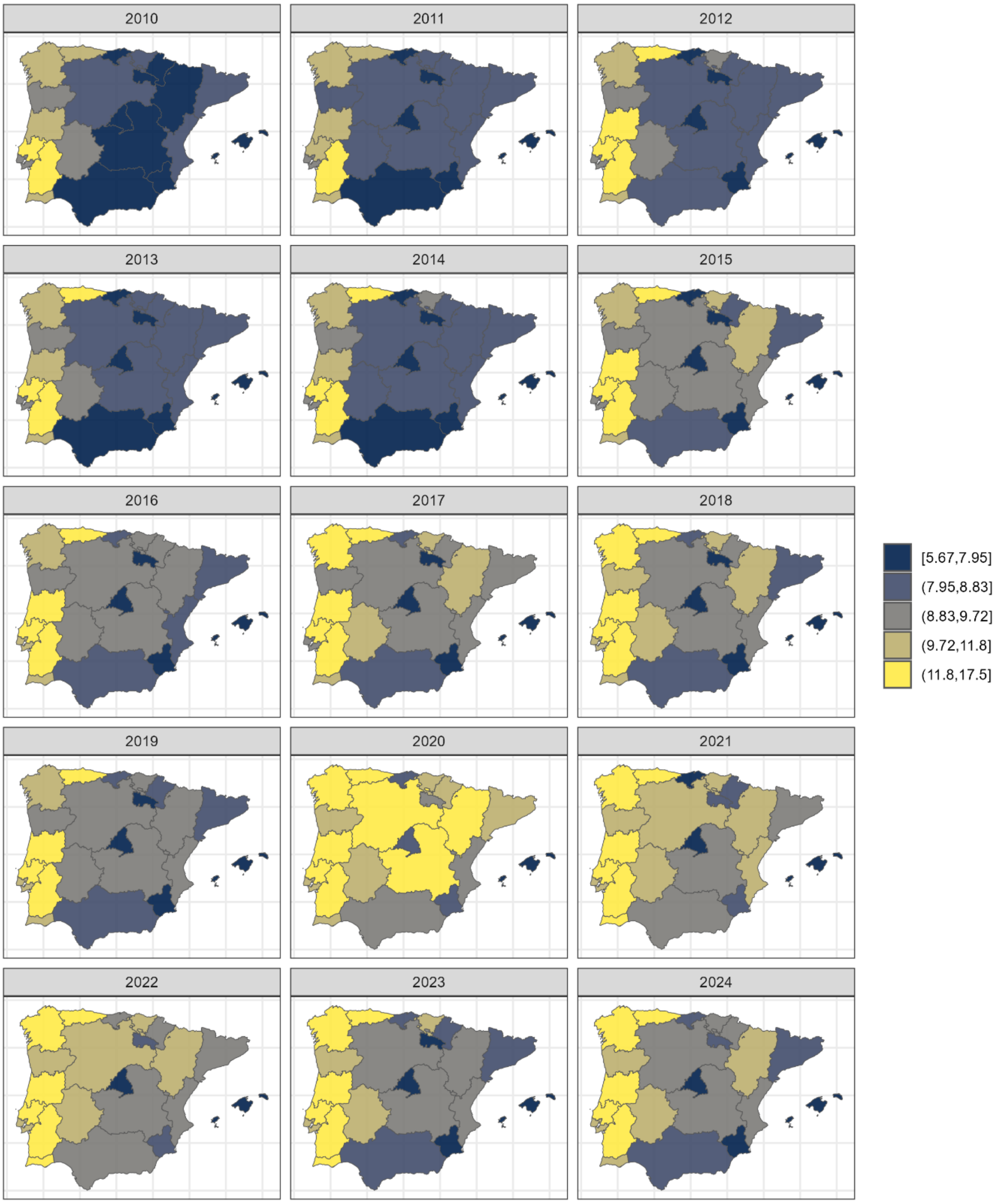

The authors should also present the mortality rate rather than the raw number of deaths.

Authors' reply: We have now modified Table S1 to include the mean mortality rate:

Portugal					
NUTSII	Number of deaths		Population	Mortality rate	Area
	Total	Mean		per 1,000	in km^2
Alentejo	111,975	7,465	484,580	15.41	27,239
Algarve	77,698	5,180	459,556	11.27	4,974
Área Metropolitana de Lisboa	425,492	28,366	2,855,491	9.93	2,844
Centro	313,639	20,909	1,667,830	12.54	23,233
Norte	525,329	35,022	3,625,496	9.66	21,256
Oeste e Vale do Tejo	160,695	10,713	841,141	12.74	9,197
Spain					
Andalucía	1,075,186	71,679	8,437,572	8.50	87,577
Aragón	187,832	12,522	1,331,353	9.41	47,660
Cantabria	70,062	4,670	586,126	7.97	5,325
Castilla y León	337,767	22,518	2,443,829	9.21	94,076
Castilla-La Mancha	285,226	19,015	2,067,634	9.20	79389
Cataluña	985,929	65,729	7,617,136	8.63	32,073
Comunidad Foral de Navarra	84,823	5,655	650,731	8.69	10,385
Comunidad Valenciana	674,300	44,953	5,038,073	8.92	23, 277
Comunidad de Madrid	626,156	41,744	6,580,400	6.34	8,010
Extremadura	156,185	10,412	1,077,873	9.66	41,604
Galicia	475,719	31,715	2,722,591	11.65	29,558
Illes Balears	126,326	8,422	1,146,807	7.34	5,010
La Rioja	36,650	2,443	318,256	7.68	5,045
País Vasco	316,036	21,069	2,193,659	9.60	7,202
Principado de Asturias	197,776	13,185	1,035,540	12.73	10,597
Región de Murcia	167,938	11,196	1,493,617	7.50	11,310

Additional contextual statistics in the table — for example, GDP per capita — would have been useful, but the authors have added a scatterplot of region-level excess mortality

against GDP per capita (supplementary figure S7), and find no correlation pattern via visual inspection. So, this may address that.

Authors' reply: The generic patterns of mortality rates as shown in the new figure, do not match the relative excess all-cause mortality as shown on Fig. 2.

We have now added one sentence citing Figure S1 in the main manuscript, page 3, lines 68-70

Online Supplement Figure S1 shows the annual mortality rates per 1,000 population across NUTS2 regions between 2010 and 2024, with the highest rates occurring during the COVID-19 pandemic.

Reviewer 2

I co-reviewed this manuscript with one of the reviewers who provided the listed reports. This is part of the Nature Communications initiative to facilitate training in peer review and to provide appropriate recognition for Early Career Researchers who co-review manuscripts.

Authors’ reply: Thank you, see our comments above.

Reviewer 3

Thank you for a strong revision. A couple small comments:

- Do nursing care facilities in Spain generally have onsite backup generators? This is common in some other high-income countries but would be helpful context. Or perhaps these are more common in Portugal.

Authors' reply: We thank the reviewer for this question. We did not identify a source that would allow us to state, at national level, that nursing care facilities in Spain or Portugal generally have onsite backup generators. We therefore prefer not to make a definitive statement in the manuscript, but we agree that variation in backup power capacity across nursing care facilities could be relevant context and now note this more cautiously in the Discussion, page 6, lines 153-156.

We did not find comparable effects in Portugal, which may reflect differences in blackout duration, baseline frailty, health-system and infrastructure conditions, and organisational features of elderly care, including preparedness measures such as backup power capacity.

- Regarding circulation of respiratory viruses, the new sentence is helpful. I wondered if including these data might improve model fit, as we have seen this to be the case in our large-scale epidemiologic studies.

Authors' reply: We thank the reviewer for this comment. We agree that incorporating indicators of respiratory virus circulation may improve model fit, especially during periods of substantial influenza or respiratory syncytial virus circulation. In the present study, however, the blackout occurred in late April 2025, when respiratory virus activity in Spain and Portugal is usually minimal. Previous evidence indicates that respiratory syncytial virus circulation in these countries is largely concentrated before week 14 of the year (https://www.eurosurveillance.org/content/10.2807/1560-7917.ES.2022.27.16.2100619). Moreover, our review of routine surveillance data for April 2025 found no evidence of unusual respiratory syncytial virus, influenza and COVID-19 activity in Spain and Portugal, Online Supplement Figure S10. We therefore expect that the inclusion of respiratory virus

covariates would yield only marginal gains in model performance during the study period and decided not to include them, pages 7-8, lines 205-210.

Although respiratory virus circulation can influence mortality, the blackout occurred outside the usual respiratory syncytial virus season in Spain and Portugal (https://www.eurosurveillance.org/content/10.2807/1560-7917.ES.2022.27.16.2100619), and surveillance data indicated no unusual respiratory syncytial virus, influenza or COVID-19 activity during April 2025 (Online Supplement Figure S10), making concurrent epidemic activity an unlikely explanation for the observed patterns and suggesting limited gains in predictive performance from including these covariates.

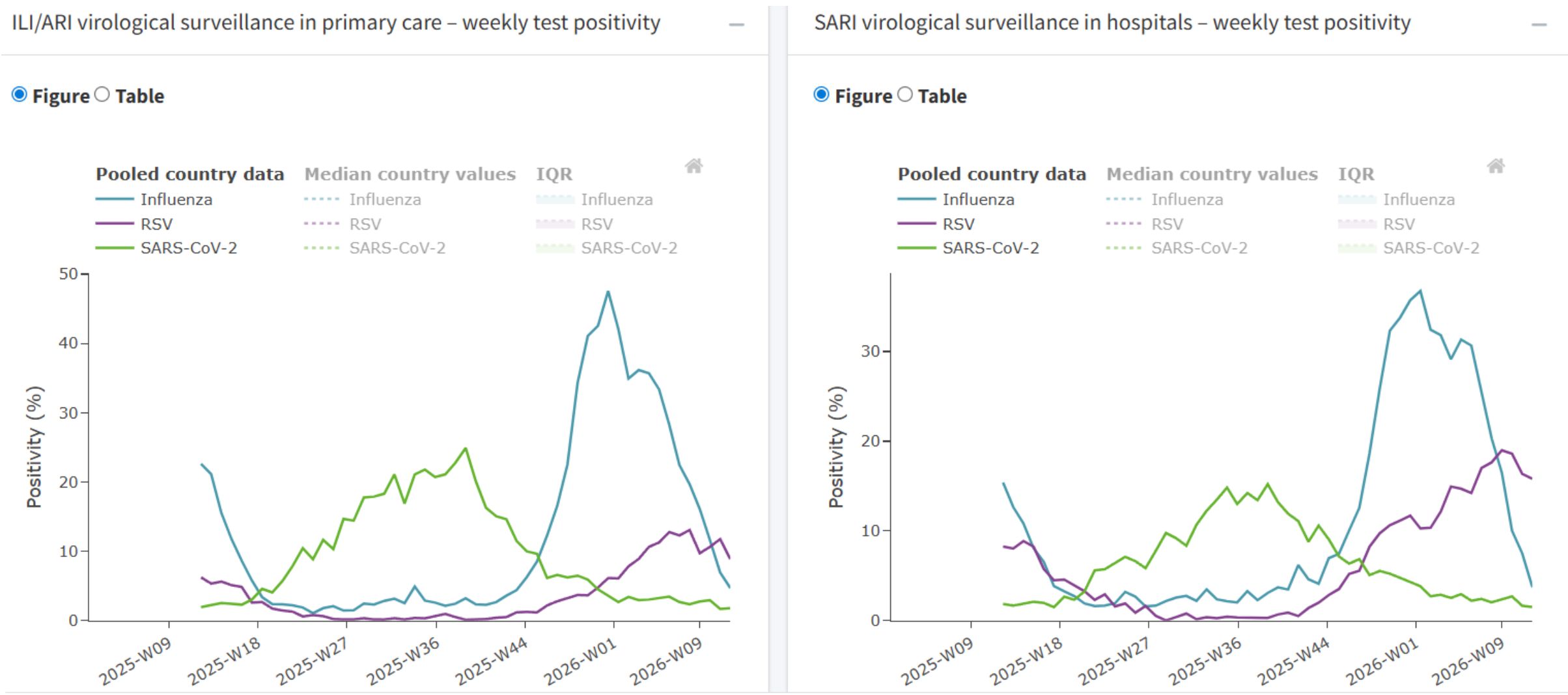

We also note that the aim of the modelling exercise was to generate predictions only one week ahead. The best-performing model included a second-order random walk and showed overall bias that was zero or close to zero across all age and sex groups when predicting sequential one-week periods in 2025 prior to the blackout. Given this short prediction horizon, temporal information from preceding weeks would be expected to capture much of the effect of any ongoing respiratory virus activity on mortality, page 8, lines 210-211.

In addition, given the one-week-ahead prediction horizon, mortality dynamics captured by the second-order random walk would likely account for much of the effect of any ongoing respiratory virus activity.

-Abstract: "Regional differences could suggest varying healthcare resilience." This is quite speculative for an abstract. I might also include "or variation in outage severity." Or cut this and add something else.

Authors' reply: We have now reformulated, see abstract on page 1.

We observed regional differences, but their drivers remain uncertain and may include variation in outage severity, underlying vulnerabilities, or local health-system and infrastructure conditions.

I have no reviewed the code.

Authors' reply: Thank you for the clarification.

Reviewer 4

Accept as it is

Authors' reply: Thank you.

Review Report

The paper studies the effect of a 10-hour blackout across mainland Spain and Portugal. It utilizes three different Poisson regression specifications trained on the data from 2010-2025 (up until the week of the blackout) to predict mortality rates, and then chooses specification (M3) as the best of their three specifications based on the metrics of bias, coverage, and mean squared error. Authors use the predictions by this model as counterfactuals and compare them with the observed mortality. The difference in mortality is associated with the effects of the blackout. Authors disaggregate mortality rates for each country by sex, age groups (0-64, 65-85, and 85+), and by lag periods: lag 0 defined as the day of the blackout, lag 0-2 days, and lag 0-6 days.

Significance

This paper addresses a public health question with limited prior evidence: the impact of blackouts on mortality. Given that extreme climate events are increasing and have the potential to cause blackouts, it is important to understand how such events affect public health to inform efficient policy responses. Unfortunately, the study does not investigate the underlying mechanisms ("how"), focusing solely on changes in mortality rates.

The paper offers a narrow empirical result in one region of the world from one particular event. Is that of sufficient impact for a broad interdisciplinary journal? Given data, the methodology is certainly replicable elsewhere. Maybe the authors can think about adding a paragraph to discuss its applicability elsewhere and for future studies.

In general, the narrative lacks a description of the methods used by other studies (which are references first in the Results, which is rather late) that have estimated mortality from power outages. The authors claim the methodology is their main novelty/contribution – if so, they should justify this against previous used methodologies.

Similarly, the authors claim their method has been used in other Covid studies. Can the authors elaborate on how other studies used their method, and why that's relevant to this study

Other Comments

1. Data Description
   The authors should provide summary statistics at the NUTS2 region level, including geographic size, population distribution, and observed mortality rates. Understanding these metrics may help clarify the heterogeneity in blackout effects.

2. Results
   Authors summarize their main findings in Figure 1 and Table 1 of the paper. Excess mortality is reported only for Spain (lag 0-2 days). Similar effects are not observed across the board which the authors need to explain. In particular,
   a. Spain:
      i. **Lag 0 days:** +14 deaths (95% CrI –59 to +80), +29 females, –15 males — all not statistically significant.
      ii. **Lag 0-2 days**: 167 (95% CrI +28 to +300), mainly from >84 years group (+99), especially females (+98).
      iii. **Lag 0-6 days**: –72 (95% CrI –202 to +319), inconclusive; still mainly **driven by >84 females**. The spike observed in lag 0-2 days disappears. If the blackout had caused mortality to spike, we might expect elevated cumulative deaths over the full week — unless vulnerable individuals died earlier than they would have, leading to fewer deaths later in the week. But this needs to be explained.
      iv. Sex differences
      The authors should explain the peculiarly high mortality among females compared to males in Spain. This contrasts with Portugal, where estimates for females are -20, -23, and -52, and males are -12, +28, +34 for the lag 0, lag0-2, and lag0-6 days, respectively.
   b. Portugal
      i. **Lag 0 days**: –32 (95% CrI –71 to +4), not statistically significant. **Though statistically significant reduced mortality is observed for >84**. (–28; 95% CrI –54 to –4), which is counterintuitive given the vulnerability of the age group.
      ii. **Lag 0-2 days**: +6 (95% CrI –65 to +73), inconclusive. Though a slight increase for 0-64 years (+21; 95% CrI -1, +41 and a slight decrease for >84 years (-17; 95%CrI -62, +27).
      iii. **Lag 0-6 days**: –19 (95% CrI –154 to +94), inconclusive.

   Mortality patterns are inconsistent across countries, sexes, age groups, and lag periods. In the absence of careful explanation, the results could reflect random fluctuations. Addressing point 3 below could help partially explain these patterns.

3. Data Limitations and Confounding Concerns
   a. No cause-of-death data utilized, which could be helpful in linking blackout effects to mortality (e.g., vulnerable individuals requiring constant care via electric medical equipment).

   b. The death records used by the authors lack the exact date of death and may include reporting delays, leading to possible misclassification of day-of-event deaths. Even a one-day delay could mean that deaths recorded on the day of the blackout occurred the previous day. The authors could partially address points 3a and 3b by triangulating their results using a subsample of records with precise cause and day of death (for example, via data from regional hospitals).
   c. In lines 94–96, the authors attribute >84 female excess mortality in Spain to the vulnerability of certain care settings (outpatient medical services, domiciliary nursing, and care homes). However, similar effects for >84 males are absent (+2; 95% CrI -56, +60).
   d. In lines 96-98, the authors cite that differences in the health care systems of Spain and Portugal are a partial explanation for the increased mortality in Spain. The authors indicate that the Spanish health care system is marked by regional disparities. Is there a documented difference in the performance of these healthcare systems across regions, and does that correlate to the authors' findings of mortality rates in different regions in Spain?
   e. In lines 100-101, the authors “found weak evidence of excess mortality among working-age males in Portugal, likely reflecting a modest rise in accidental deaths associated with the blackout.” A similar effect is not observed for working-age males in Spain. Why did a similar increase not happen in Spain?

4. Presentation
   a. Line 122, replace ‘out’ with ‘our’.
   b. A visual representation of Table 1 would help grasp the findings more conveniently. See the example below (may not accurately represent all the mortality numbers).

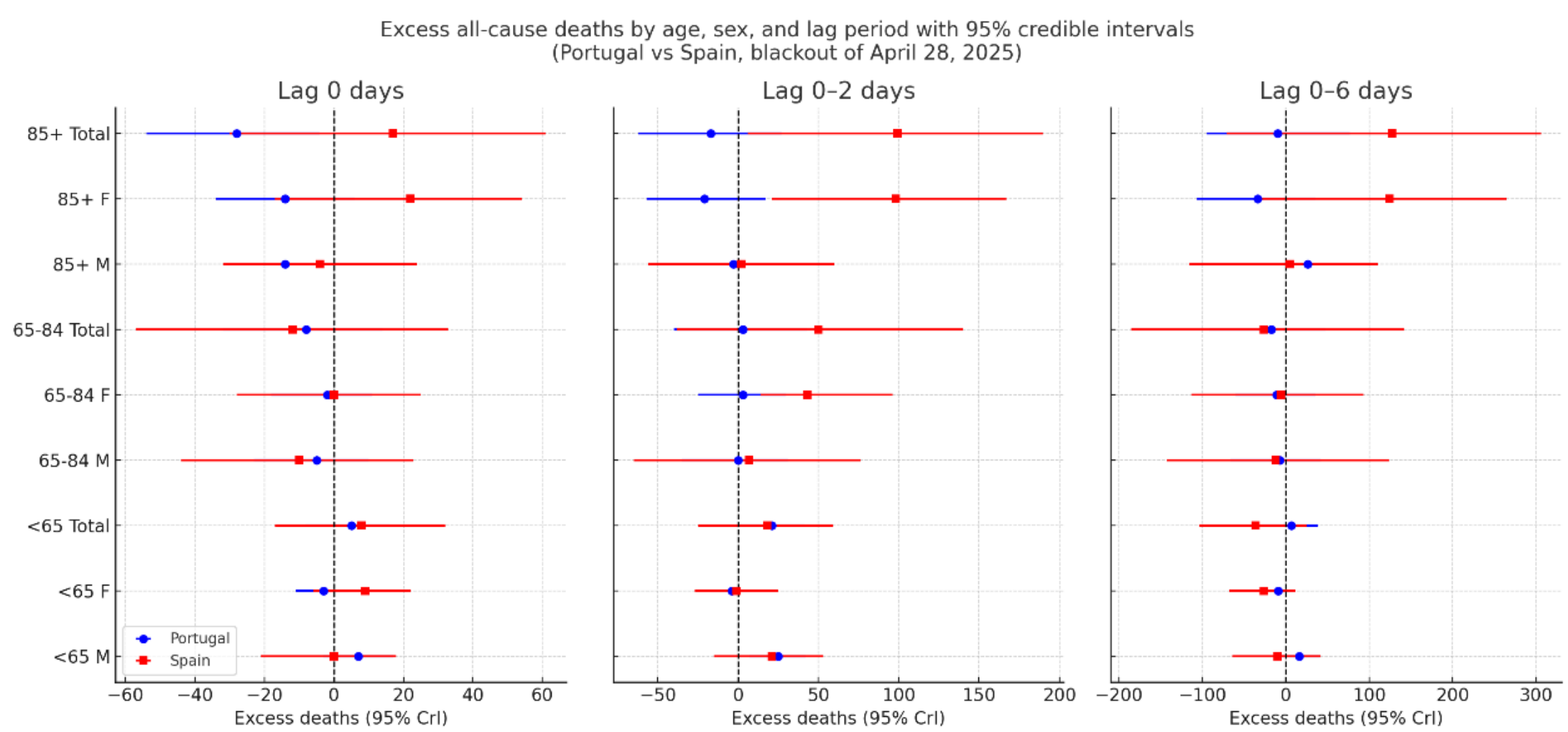

5. Model Specifications
   The authors should briefly explain why the specific model specifications were used and why M3 performed slightly better than M2 and M1. M3 utilizes a second-order random walk, which provides a smooth data-driven curve that avoids overfitting to small random noise. This is appropriate for mortality trends that change gradually without erratic week-to-week jumps.

Review of "Excess mortality attributable to the 2025 Iberian Peninsula blackout"

26th November

Summary

In this manuscript, the authors present the first systematic assessment of all-cause excess mortality associated with the April 2025 blackout that affected mainland Portugal and Spain. Using daily mortality and population data at the NUTS2 level, stratified by age and sex, the authors fit Bayesian spatiotemporal models to estimate counterfactual expected deaths for the blackout week. The modelling framework incorporates distributed lag non-linear temperature effects, long-term and seasonal trends, day-of-week patterns, holidays, and regional random effects, and is implemented using INLA. Excess mortality is then quantified by comparing observed deaths with the model-based predictions across several predefined lag windows.

The results suggest no clear excess mortality on the day of the blackout, but indicate a short-term increase in Spain over the subsequent two days, particularly among women aged 85+. No consistent signal is found in Portugal. The authors interpret these patterns as evidence of heterogeneous vulnerability to blackout-related disruptions, with possible implications for healthcare resilience.

The topic is timely and relevant, and the modelling effort is substantial. The work has potential value for understanding health impacts of large-scale infrastructural failures. However, several aspects of the study design, model specification, and interpretation would benefit from further clarification and refinement before the conclusions can be fully supported.

The paper

Major comments

1. **Air pollution as an omitted confounder (lines 163 -)**
   The models adjust for temperature using a DLNM framework, but do not include any air-pollution covariates. Given the well-established short-term effects of $PM_{2.5}$, PM_{10} and NO_2 on daily mortality,

and the fact that blackouts may temporarily alter emissions through generator use, traffic disruption or indoor combustion, a clarification would be welcome. Even if pollution data were unavailable or too coarse, briefly discussing the potential for confounding, or providing a sensitivity assessment, would help strengthen the interpretation of the excess mortality estimates.

2. **Socioeconomic determinants and baseline vulnerability**

   Given the clear heterogeneity in excess mortality by region and by age–sex group, it would be helpful to discuss more explicitly the role of underlying socioeconomic conditions.

3. **Clarification of national holidays included in the model (line 164)**

   The manuscript mentions the inclusion of national holidays as a binary covariate, but it is not clear which specific holidays were incorporated for each country and whether regional holidays were also considered. This detail matters because holiday calendars could differ across autonomous communities in Spain, and in 2025, 28 April was a public holiday in the Valencian Community. It would be helpful if the authors could clarify.

4. **Specification of the covariate $X_{2,mt}$ in Model 2 (line 170)**

   In Model 2, $X_{2,mt}$ is described as "the week and the year", but this definition is ambiguous. It would be helpful if the authors could specify whether $X_{2,mt}$ represents a linear trend in calendar time, a combination of week number and year indicator, or some other coding scheme. Because this term interacts with seasonality and long-term trends, a clearer description would assist readers in understanding how Model 2 differs structurally from Models 1 and 3.

5. **Justification for knot placement in the B-spline basis (line 174)**

   The DLNM uses quadratic B-splines with internal knots placed at 10°C, 18°C and 27°C, but the manuscript does not discuss why these particular values were selected. While these temperatures may be reasonable in the Iberian context, the modelling of temperature–mortality relationships is sensitive to knot choice. A short justification—empirical, climatological, or based on prior literature—or a brief sensitivity check would strengthen confidence in the robustness of the temperature adjustment.

6. **Spatial structure of regional random effects (line 177)**

   The manuscript models the regional random effects z_s as i.i.d. normal, arguing that this choice is adequate given the relatively large size of NUTS2 regions. However, neighbouring regions are likely to share important unmeasured determinants of mortality (healthcare organisation, socio-demographic profiles, infrastructure, climate), so some degree of spatial correlation in the residuals would be plausible. I would encourage the authors either to provide more justification for the i.i.d. assumption (e.g., via residual diagnostics such as Moran's I) or, if feasible, to report a brief sensitivity analysis using a structured spatial prior (e.g., CAR/BYM2). This would help to assess whether the estimated excess mortality is robust to alternative assumptions about spatial dependence.

7. **Clarification of the prior on the intercept (line 179)**
   The manuscript states that the intercept is assigned a "normal prior with mean zero and zero precision". Since in INLA this parametrisation corresponds to an *improper uniform prior* on the real line (i.e., a flat prior implemented via infinite variance), it may be clearer to describe it explicitly as such. I would encourage the authors to replace the current wording with a formulation such as "an improper uniform prior for the intercept", which more accurately reflects the prior structure and avoids potential confusion for readers unfamiliar with INLA's internal parametrisation.

8. **PC priors for random-effect hyperparameters (LINE 180–182)**
   The description of the penalised complexity (PC) priors is rather brief. Given the number of latent components in the model, each of which introduces its own precision hyperparameter (e.g., the RW2 temporal trend, seasonal effects, and the DLNM temperature basis), it would be helpful for the authors to provide a clearer and more comprehensive account of all hyperparameters involved and the specific PC priors assigned to them. In particular, RW2 terms generate a precision parameter with its own PC prior, while spline-based components may rely on different penalisation structures. If any SPDE-based representation or other sparse formulations were used for the B-splines, the associated PC priors may differ accordingly. A more explicit description of how each hyperparameter is defined and penalised would improve transparency and help readers understand the regularisation applied throughout the model.

Minor Comments

- **Typographical error in affiliation (line 3)**
  "Evnironment" should be corrected to "Environment".

- **Consistency in spelling: "behavior/behaviour" and "organisation/organization"**
  The manuscript mixes British and American spellings. It may be preferable to standardise this.

- **Unclear reference to "the model" in the Results (lines 64–75)**
  At several points, the text refers simply to "the model" without specifying which of the three candidate models is being discussed. Adding the model number would avoid ambiguity.

- **Ambiguity in "the week of the blackout" (LINE 49)**
  Specify that this refers to 28 April–4 May 2025 for clarity.

- **Typographical issues: duplicated commas and inconsistent spacing (e.g., lines 96–100)**
  A quick proofread would resolve minor spacing inconsistencies, such as double spaces after commas.

- **Acronym definition (line 111)**
  DLNM (Distributed Lag Non-linear Model) is used before being spelled out. Define at first occurrence for clarity to non-specialist readers.

- **Typographical error (line 122)**
  The text reads "out study lacks detailed data…", which should be corrected to "our study".

- **Non-functional GitHub link (lines 209–211)**
  The manuscript provides a GitHub repository for reproducing the analysis (https://github.com/jriou/blackout-burden), but the link returns an error.

Decision: major revision
